# Measurement of multidifferential cross sections for dijet production in proton–proton collisions at $\sqrt{s} = 13\,\text {Te}\hspace{-.08em}\text {V}$

**DOI:** 10.1140/epjc/s10052-024-13606-8

**Published:** 2025-01-24

**Authors:** CMS Collaboration, A. Hayrapetyan, A. Tumasyan, W. Adam, J. W. Andrejkovic, T. Bergauer, S. Chatterjee, K. Damanakis, M. Dragicevic, A. Escalante Del Valle, P. S. Hussain, M. Jeitler, N. Krammer, L. Lechner, D. Liko, I. Mikulec, J. Schieck, R. Schöfbeck, D. Schwarz, M. Sonawane, S. Templ, W. Waltenberger, C.-E. Wulz, M. R. Darwish, T. Janssen, T. Kello, P. Van Mechelen, E. S. Bols, J. D’Hondt, A. De Moor, M. Delcourt, H. El Faham, S. Lowette, I. Makarenko, A. Morton, D. Müller, A. R. Sahasransu, S. Tavernier, S. Van Putte, D. Vannerom, B. Clerbaux, S. Dansana, G. De Lentdecker, L. Favart, D. Hohov, J. Jaramillo, K. Lee, M. Mahdavikhorrami, A. Malara, S. Paredes, L. Pétré, N. Postiau, L. Thomas, M. Vanden Bemden, C. Vander Velde, P. Vanlaer, M. De Coen, D. Dobur, J. Knolle, L. Lambrecht, G. Mestdach, C. Rendón, A. Samalan, K. Skovpen, M. Tytgat, N. Van Den Bossche, B. Vermassen, L. Wezenbeek, A. Benecke, G. Bruno, F. Bury, C. Caputo, C. Delaere, I. S. Donertas, A. Giammanco, K. Jaffel, Sa. Jain, V. Lemaitre, J. Lidrych, P. Mastrapasqua, K. Mondal, T. T. Tran, S. Wertz, G. A. Alves, E. Coelho, C. Hensel, A. Moraes, P. Rebello Teles, W. L. Aldá Júnior, M. Alves Gallo Pereira, M. Barroso Ferreira Filho, H. Brandao Malbouisson, W. Carvalho, J. Chinellato, E. M. Da Costa, G. G. Da Silveira, D. De Jesus Damiao, V. Dos Santos Sousa, S. Fonseca De Souza, J. Martins, C. Mora Herrera, K. Mota Amarilo, L. Mundim, H. Nogima, A. Santoro, S. M. Silva Do Amaral, A. Sznajder, M. Thiel, A. Vilela Pereira, C. A. Bernardes, L. Calligaris, T. R. Fernandez Perez Tomei, E. M. Gregores, P. G. Mercadante, S. F. Novaes, B. Orzari, Sandra S. Padula, A. Aleksandrov, G. Antchev, R. Hadjiiska, P. Iaydjiev, M. Misheva, M. Shopova, G. Sultanov, A. Dimitrov, T. Ivanov, L. Litov, B. Pavlov, P. Petkov, A. Petrov, E. Shumka, S. Keshri, S. Thakur, T. Cheng, Q. Guo, T. Javaid, M. Mittal, L. Yuan, G. Bauer, Z. Hu, K. Yi, G. M. Chen, H. S. Chen, M. Chen, F. Iemmi, C. H. Jiang, A. Kapoor, H. Liao, Z.-A. Liu, F. Monti, R. Sharma, J. N. Song, J. Tao, C. Wang, J. Wang, H. Zhang, A. Agapitos, Y. Ban, A. Levin, C. Li, Q. Li, X. Lyu, Y. Mao, S. J. Qian, X. Sun, D. Wang, H. Yang, M. Lu, Z. You, N. Lu, X. Gao, D. Leggat, H. Okawa, Y. Zhang, Z. Lin, C. Lu, M. Xiao, C. Avila, D. A. Barbosa Trujillo, A. Cabrera, C. Florez, J. Fraga, J. A. Reyes Vega, J. Mejia Guisao, F. Ramirez, M. Rodriguez, J. D. Ruiz Alvarez, D. Giljanovic, N. Godinovic, D. Lelas, A. Sculac, M. Kovac, T. Sculac, P. Bargassa, V. Brigljevic, B. K. Chitroda, D. Ferencek, S. Mishra, A. Starodumov, T. Susa, A. Attikis, K. Christoforou, S. Konstantinou, J. Mousa, C. Nicolaou, F. Ptochos, P. A. Razis, H. Rykaczewski, H. Saka, A. Stepennov, M. Finger, M. Finger, A. Kveton, E. Ayala, E. Carrera Jarrin, A. A. Abdelalim, E. Salama, M. A. Mahmoud, Y. Mohammed, K. Ehataht, M. Kadastik, T. Lange, S. Nandan, C. Nielsen, J. Pata, M. Raidal, L. Tani, C. Veelken, H. Kirschenmann, K. Osterberg, M. Voutilainen, S. Bharthuar, E. Brücken, F. Garcia, J. Havukainen, K. T. S. Kallonen, M. S. Kim, R. Kinnunen, T. Lampén, K. Lassila-Perini, S. Lehti, T. Lindén, M. Lotti, L. Martikainen, M. Myllymäki, M.m. Rantanen, H. Siikonen, E. Tuominen, J. Tuominiemi, P. Luukka, H. Petrow, T. Tuuva, C. Amendola, M. Besancon, F. Couderc, M. Dejardin, D. Denegri, J. L. Faure, F. Ferri, S. Ganjour, P. Gras, G. Hamel de Monchenault, V. Lohezic, J. Malcles, J. Rander, A. Rosowsky, M.Ö. Sahin, A. Savoy-Navarro, P. Simkina, M. Titov, C. Baldenegro Barrera, F. Beaudette, A. Buchot Perraguin, P. Busson, A. Cappati, C. Charlot, F. Damas, O. Davignon, B. Diab, G. Falmagne, B. A. Fontana Santos Alves, S. Ghosh, R. Granier de Cassagnac, A. Hakimi, B. Harikrishnan, G. Liu, J. Motta, M. Nguyen, C. Ochando, L. Portales, R. Salerno, U. Sarkar, J. B. Sauvan, Y. Sirois, A. Tarabini, E. Vernazza, A. Zabi, A. Zghiche, J.-L. Agram, J. Andrea, D. Apparu, D. Bloch, J.-M. Brom, E. C. Chabert, C. Collard, U. Goerlach, C. Grimault, A.-C. Le Bihan, P. Van Hove, S. Beauceron, B. Blancon, G. Boudoul, N. Chanon, J. Choi, D. Contardo, P. Depasse, C. Dozen, H. El Mamouni, J. Fay, S. Gascon, M. Gouzevitch, C. Greenberg, G. Grenier, B. Ille, I. B. Laktineh, M. Lethuillier, L. Mirabito, S. Perries, M. Vander Donckt, P. Verdier, J. Xiao, G. Adamov, I. Lomidze, Z. Tsamalaidze, V. Botta, L. Feld, K. Klein, M. Lipinski, D. Meuser, A. Pauls, N. Röwert, M. Teroerde, S. Diekmann, A. Dodonova, N. Eich, D. Eliseev, M. Erdmann, P. Fackeldey, B. Fischer, T. Hebbeker, K. Hoepfner, F. Ivone, M. Y. Lee, L. Mastrolorenzo, M. Merschmeyer, A. Meyer, S. Mondal, S. Mukherjee, D. Noll, A. Novak, F. Nowotny, A. Pozdnyakov, Y. Rath, W. Redjeb, F. Rehm, H. Reithler, A. Schmidt, S. C. Schuler, A. Sharma, A. Stein, F. Torres Da Silva De Araujo, L. Vigilante, S. Wiedenbeck, S. Zaleski, C. Dziwok, G. Flügge, W. Haj Ahmad, T. Kress, A. Nowack, O. Pooth, A. Stahl, T. Ziemons, A. Zotz, H. Aarup Petersen, M. Aldaya Martin, J. Alimena, S. Amoroso, Y. An, S. Baxter, M. Bayatmakou, H. Becerril Gonzalez, O. Behnke, S. Bhattacharya, F. Blekman, K. Borras, D. Brunner, A. Campbell, A. Cardini, C. Cheng, F. Colombina, S. Consuegra Rodríguez, G. Correia Silva, M. De Silva, G. Eckerlin, D. Eckstein, L. I. Estevez Banos, O. Filatov, E. Gallo, A. Geiser, A. Giraldi, G. Greau, V. Guglielmi, M. Guthoff, A. Jafari, N. Z. Jomhari, B. Kaech, M. Kasemann, H. Kaveh, C. Kleinwort, R. Kogler, M. Komm, D. Krücker, W. Lange, D. Leyva Pernia, K. Lipka, W. Lohmann, R. Mankel, I.-A. Melzer-Pellmann, M. Mendizabal Morentin, J. Metwally, A. B. Meyer, G. Milella, M. Mormile, A. Mussgiller, A. Nürnberg, Y. Otarid, D. Pérez Adán, E. Ranken, A. Raspereza, B. Ribeiro Lopes, J. Rübenach, A. Saggio, M. Scham, V. Scheurer, S. Schnake, P. Schütze, C. Schwanenberger, M. Shchedrolosiev, R. E. Sosa Ricardo, L. P. Sreelatha Pramod, D. Stafford, F. Vazzoler, A. Ventura Barroso, R. Walsh, Q. Wang, Y. Wen, K. Wichmann, L. Wiens, C. Wissing, S. Wuchterl, Y. Yang, A. Zimermmane Castro Santos, A. Albrecht, S. Albrecht, M. Antonello, S. Bein, L. Benato, M. Bonanomi, P. Connor, K. De Leo, M. Eich, K. El Morabit, Y. Fischer, A. Fröhlich, C. Garbers, E. Garutti, A. Grohsjean, M. Hajheidari, J. Haller, A. Hinzmann, H. R. Jabusch, G. Kasieczka, P. Keicher, R. Klanner, W. Korcari, T. Kramer, V. Kutzner, F. Labe, J. Lange, A. Lobanov, C. Matthies, A. Mehta, L. Moureaux, M. Mrowietz, A. Nigamova, Y. Nissan, A. Paasch, K. J. Pena Rodriguez, T. Quadfasel, M. Rieger, D. Savoiu, J. Schindler, P. Schleper, M. Schröder, J. Schwandt, M. Sommerhalder, H. Stadie, G. Steinbrück, A. Tews, M. Wolf, S. Brommer, M. Burkart, E. Butz, T. Chwalek, A. Dierlamm, A. Droll, N. Faltermann, M. Giffels, A. Gottmann, F. Hartmann, M. Horzela, U. Husemann, M. Klute, R. Koppenhöfer, M. Link, A. Lintuluoto, S. Maier, S. Mitra, Th. Müller, M. Neukum, M. Oh, G. Quast, K. Rabbertz, I. Shvetsov, H. J. Simonis, N. Trevisani, R. Ulrich, J. van der Linden, R. F. Von Cube, M. Wassmer, S. Wieland, R. Wolf, S. Wunsch, X. Zuo, G. Anagnostou, P. Assiouras, G. Daskalakis, A. Kyriakis, A. Stakia, D. Karasavvas, P. Kontaxakis, G. Melachroinos, A. Panagiotou, I. Papavergou, I. Paraskevas, N. Saoulidou, K. Theofilatos, E. Tziaferi, K. Vellidis, I. Zisopoulos, G. Bakas, T. Chatzistavrou, G. Karapostoli, K. Kousouris, I. Papakrivopoulos, E. Siamarkou, G. Tsipolitis, A. Zacharopoulou, K. Adamidis, I. Bestintzanos, I. Evangelou, C. Foudas, P. Gianneios, C. Kamtsikis, P. Katsoulis, P. Kokkas, P. G. Kosmoglou Kioseoglou, N. Manthos, I. Papadopoulos, J. Strologas, M. Bartók, C. Hajdu, D. Horvath, F. Sikler, V. Veszpremi, M. Csanád, K. Farkas, M. M. A. Gadallah, P. Major, K. Mandal, G. Pásztor, A. J. Rádl, O. Surányi, G. I. Veres, G. Bencze, S. Czellar, J. Karancsi, J. Molnar, Z. Szillasi, P. Raics, B. Ujvari, G. Zilizi, T. Csorgo, F. Nemes, T. Novak, J. Babbar, S. Bansal, S. B. Beri, V. Bhatnagar, G. Chaudhary, S. Chauhan, N. Dhingra, R. Gupta, A. Kaur, A. Kaur, H. Kaur, M. Kaur, S. Kumar, P. Kumari, M. Meena, K. Sandeep, T. Sheokand, J. B. Singh, A. Singla, A. Ahmed, A. Bhardwaj, A. Chhetri, B. C. Choudhary, A. Kumar, M. Naimuddin, K. Ranjan, S. Saumya, S. Baradia, S. Barman, S. Bhattacharya, D. Bhowmik, S. Dutta, S. Dutta, B. Gomber, P. Palit, G. Saha, B. Sahu, S. Sarkar, P. K. Behera, S. C. Behera, S. Chatterjee, P. Jana, P. Kalbhor, J. R. Komaragiri, D. Kumar, M. Mohammad Mobassir Ameen, A. Muhammad, L. Panwar, R. Pradhan, P. R. Pujahari, N. R. Saha, A. Sharma, A. K. Sikdar, S. Verma, T. Aziz, I. Das, S. Dugad, M. Kumar, G. B. Mohanty, P. Suryadevara, A. Bala, S. Banerjee, M. Guchait, S. Karmakar, S. Kumar, G. Majumder, K. Mazumdar, S. Mukherjee, A. Thachayath, S. Bahinipati, A. K. Das, C. Kar, D. Maity, P. Mal, T. Mishra, V. K. Muraleedharan Nair Bindhu, K. Naskar, A. Nayak, P. Saha, S. K. Swain, S. Varghese, D. Vats, A. Alpana, S. Dube, B. Kansal, A. Laha, S. Pandey, A. Rastogi, S. Sharma, H. Bakhshiansohi, E. Khazaie, M. Zeinali, S. Chenarani, S. M. Etesami, M. Khakzad, M. Mohammadi Najafabadi, M. Grunewald, M. Abbrescia, R. Aly, C. Aruta, A. Colaleo, D. Creanza, B. D’Anzi, N. De Filippis, M. De Palma, A. Di Florio, W. Elmetenawee, F. Errico, L. Fiore, G. Iaselli, G. Maggi, M. Maggi, V. Mastrapasqua, S. My, S. Nuzzo, A. Pellecchia, A. Pompili, G. Pugliese, R. Radogna, D. Ramos, A. Ranieri, L. Silvestris, Ü. Sözbilir, A. Stamerra, R. Venditti, P. Verwilligen, A. Zaza, G. Abbiendi, C. Battilana, D. Bonacorsi, L. Borgonovi, P. Capiluppi, A. Castro, F. R. Cavallo, M. Cuffiani, G. M. Dallavalle, T. Diotalevi, F. Fabbri, A. Fanfani, D. Fasanella, P. Giacomelli, L. Giommi, C. Grandi, L. Guiducci, S. Lo Meo, L. Lunerti, S. Marcellini, G. Masetti, F. L. Navarria, A. Perrotta, F. Primavera, A. M. Rossi, T. Rovelli, G. P. Siroli, S. Costa, A. Di Mattia, R. Potenza, A. Tricomi, C. Tuve, G. Barbagli, G. Bardelli, B. Camaiani, A. Cassese, R. Ceccarelli, V. Ciulli, C. Civinini, R. D’Alessandro, E. Focardi, G. Latino, P. Lenzi, M. Lizzo, M. Meschini, S. Paoletti, G. Sguazzoni, L. Viliani, L. Benussi, S. Bianco, S. Meola, D. Piccolo, P. Chatagnon, F. Ferro, E. Robutti, S. Tosi, A. Benaglia, G. Boldrini, F. Brivio, F. Cetorelli, F. De Guio, M. E. Dinardo, P. Dini, S. Gennai, A. Ghezzi, P. Govoni, L. Guzzi, M. T. Lucchini, M. Malberti, S. Malvezzi, A. Massironi, D. Menasce, L. Moroni, M. Paganoni, D. Pedrini, B. S. Pinolini, S. Ragazzi, N. Redaelli, T. Tabarelli de Fatis, D. Zuolo, S. Buontempo, A. Cagnotta, F. Carnevali, N. Cavallo, A. De Iorio, F. Fabozzi, A. O. M. Iorio, L. Lista, P. Paolucci, B. Rossi, C. Sciacca, R. Ardino, P. Azzi, N. Bacchetta, D. Bisello, P. Bortignon, A. Bragagnolo, R. Carlin, P. Checchia, T. Dorigo, F. Gasparini, U. Gasparini, G. Grosso, M. Gulmini, L. Layer, E. Lusiani, M. Margoni, A. T. Meneguzzo, M. Migliorini, J. Pazzini, P. Ronchese, R. Rossin, F. Simonetto, G. Strong, M. Tosi, A. Triossi, H. Yarar, M. Zanetti, P. Zotto, A. Zucchetta, G. Zumerle, S. Abu Zeid, C. Aimè, A. Braghieri, S. Calzaferri, D. Fiorina, P. Montagna, V. Re, C. Riccardi, P. Salvini, I. Vai, P. Vitulo, P. Asenov, G. M. Bilei, D. Ciangottini, L. Fanò, M. Magherini, G. Mantovani, V. Mariani, M. Menichelli, F. Moscatelli, A. Piccinelli, M. Presilla, A. Rossi, A. Santocchia, D. Spiga, T. Tedeschi, P. Azzurri, G. Bagliesi, R. Bhattacharya, L. Bianchini, T. Boccali, E. Bossini, D. Bruschini, R. Castaldi, M. A. Ciocci, V. D’Amante, R. Dell’Orso, S. Donato, A. Giassi, F. Ligabue, D. Matos Figueiredo, A. Messineo, M. Musich, F. Palla, S. Parolia, G. Ramirez-Sanchez, A. Rizzi, G. Rolandi, S. Roy Chowdhury, T. Sarkar, A. Scribano, P. Spagnolo, R. Tenchini, G. Tonelli, N. Turini, A. Venturi, P. G. Verdini, P. Barria, M. Campana, F. Cavallari, L. Cunqueiro Mendez, D. Del Re, E. Di Marco, M. Diemoz, E. Longo, P. Meridiani, J. Mijuskovic, G. Organtini, F. Pandolfi, R. Paramatti, C. Quaranta, S. Rahatlou, C. Rovelli, F. Santanastasio, L. Soffi, R. Tramontano, N. Amapane, R. Arcidiacono, S. Argiro, M. Arneodo, N. Bartosik, R. Bellan, A. Bellora, C. Biino, N. Cartiglia, M. Costa, R. Covarelli, N. Demaria, L. Finco, M. Grippo, B. Kiani, F. Legger, F. Luongo, C. Mariotti, S. Maselli, A. Mecca, E. Migliore, M. Monteno, R. Mulargia, M. M. Obertino, G. Ortona, L. Pacher, N. Pastrone, M. Pelliccioni, M. Ruspa, K. Shchelina, F. Siviero, V. Sola, A. Solano, D. Soldi, A. Staiano, C. Tarricone, M. Tornago, D. Trocino, G. Umoret, A. Vagnerini, E. Vlasov, S. Belforte, V. Candelise, M. Casarsa, F. Cossutti, G. Della Ricca, G. Sorrentino, S. Dogra, C. Huh, B. Kim, D. H. Kim, J. Kim, J. Lee, S. W. Lee, C. S. Moon, Y. D. Oh, S. I. Pak, M. S. Ryu, S. Sekmen, Y. C. Yang, G. Bak, P. Gwak, H. Kim, D. H. Moon, E. Asilar, T. J. Kim, J. Park, S. Choi, S. Han, B. Hong, K. Lee, K. S. Lee, J. Lim, J. Park, S. K. Park, J. Yoo, J. Goh, H. S. Kim, Y. Kim, S. Lee, J. Almond, J. H. Bhyun, J. Choi, S. Jeon, J. Kim, J. S. Kim, S. Ko, H. Kwon, H. Lee, S. Lee, B. H. Oh, S. B. Oh, H. Seo, U. K. Yang, I. Yoon, W. Jang, D. Y. Kang, Y. Kang, D. Kim, S. Kim, B. Ko, J. S. H. Lee, Y. Lee, J. A. Merlin, I. C. Park, Y. Roh, I. J. Watson, S. Yang, S. Ha, H. D. Yoo, M. Choi, M. R. Kim, H. Lee, Y. Lee, I. Yu, T. Beyrouthy, Y. Maghrbi, K. Dreimanis, A. Gaile, G. Pikurs, A. Potrebko, M. Seidel, V. Veckalns, N. R. Strautnieks, M. Ambrozas, A. Juodagalvis, A. Rinkevicius, G. Tamulaitis, N. Bin Norjoharuddeen, I. Yusuff, Z. Zolkapli, J. F. Benitez, A. Castaneda Hernandez, H. A. Encinas Acosta, L. G. Gallegos Maríñez, M. León Coello, J. A. Murillo Quijada, A. Sehrawat, L. Valencia Palomo, G. Ayala, H. Castilla-Valdez, E. De La Cruz-Burelo, I. Heredia-De La Cruz, R. Lopez-Fernandez, C. A. Mondragon Herrera, D. A. Perez Navarro, A. Sánchez Hernández, C. Oropeza Barrera, M. Ramírez García, I. Pedraza, H. A. Salazar Ibarguen, C. Uribe Estrada, I. Bubanja, N. Raicevic, P. H. Butler, A. Ahmad, M. I. Asghar, A. Awais, M. I. M. Awan, H. R. Hoorani, W. A. Khan, V. Avati, L. Grzanka, M. Malawski, H. Bialkowska, M. Bluj, B. Boimska, M. Górski, M. Kazana, M. Szleper, P. Zalewski, K. Bunkowski, K. Doroba, A. Kalinowski, M. Konecki, J. Krolikowski, K. Pozniak, W. Zabolotny, M. Araujo, D. Bastos, C. Beirão Da Cruz E Silva, A. Boletti, M. Bozzo, P. Faccioli, M. Gallinaro, J. Hollar, N. Leonardo, T. Niknejad, M. Pisano, J. Seixas, J. Varela, P. Adzic, P. Milenovic, M. Dordevic, J. Milosevic, V. Rekovic, M. Aguilar-Benitez, J. Alcaraz Maestre, M. Barrio Luna, Cristina F. Bedoya, M. Cepeda, M. Cerrada, N. Colino, B. De La Cruz, A. Delgado Peris, D. Fernández Del Val, J. P. Fernández Ramos, J. Flix, M. C. Fouz, O. Gonzalez Lopez, S. Goy Lopez, J. M. Hernandez, M. I. Josa, J. León Holgado, D. Moran, Á. Navarro Tobar, C. Perez Dengra, A. Pérez-Calero Yzquierdo, J. Puerta Pelayo, I. Redondo, D. D. Redondo Ferrero, L. Romero, S. Sánchez Navas, L. Urda Gómez, J. Vazquez Escobar, C. Willmott, J. F. de Trocóniz, B. Alvarez Gonzalez, J. Cuevas, J. Fernandez Menendez, S. Folgueras, I. Gonzalez Caballero, J. R. González Fernández, E. Palencia Cortezon, C. Ramón Álvarez, V. Rodríguez Bouza, A. Soto Rodríguez, A. Trapote, C. Vico Villalba, P. Vischia, S. Bhowmik, S. Blanco Fernández, J. A. Brochero Cifuentes, I. J. Cabrillo, A. Calderon, J. Duarte Campderros, M. Fernandez, C. Fernandez Madrazo, G. Gomez, C. Lasaosa García, C. Martinez Rivero, P. Martinez Ruiz del Arbol, F. Matorras, P. Matorras Cuevas, E. Navarrete Ramos, J. Piedra Gomez, C. Prieels, L. Scodellaro, I. Vila, J. M. Vizan Garcia, M. K. Jayananda, B. Kailasapathy, D. U. J. Sonnadara, D. D. C. Wickramarathna, W. G. D. Dharmaratna, K. Liyanage, N. Perera, N. Wickramage, D. Abbaneo, E. Auffray, G. Auzinger, J. Baechler, D. Barney, A. Bermúdez Martínez, M. Bianco, B. Bilin, A. A. Bin Anuar, A. Bocci, E. Brondolin, C. Caillol, T. Camporesi, G. Cerminara, N. Chernyavskaya, M. Cipriani, D. d’Enterria, A. Dabrowski, A. David, A. De Roeck, M. M. Defranchis, M. Deile, M. Dobson, F. Fallavollita, L. Forthomme, G. Franzoni, W. Funk, S. Giani, D. Gigi, K. Gill, F. Glege, L. Gouskos, M. Haranko, J. Hegeman, T. James, J. Kieseler, N. Kratochwil, S. Laurila, P. Lecoq, E. Leutgeb, C. Lourenço, B. Maier, L. Malgeri, M. Mannelli, A. C. Marini, F. Meijers, S. Mersi, E. Meschi, F. Moortgat, M. Mulders, S. Orfanelli, F. Pantaleo, M. Peruzzi, A. Petrilli, G. Petrucciani, A. Pfeiffer, M. Pierini, D. Piparo, H. Qu, D. Rabady, G. Reales Gutiérrez, M. Rovere, H. Sakulin, S. Scarfi, M. Selvaggi, A. Sharma, P. Silva, P. Sphicas, A. G. Stahl Leiton, A. Steen, S. Summers, D. Treille, P. Tropea, A. Tsirou, D. Walter, J. Wanczyk, K. A. Wozniak, P. Zejdl, W. D. Zeuner, T. Bevilacqua, L. Caminada, A. Ebrahimi, W. Erdmann, R. Horisberger, Q. Ingram, H. C. Kaestli, D. Kotlinski, C. Lange, M. Missiroli, L. Noehte, T. Rohe, T. K. Aarrestad, K. Androsov, M. Backhaus, A. Calandri, K. Datta, A. De Cosa, G. Dissertori, M. Dittmar, M. Donegà, F. Eble, M. Galli, K. Gedia, F. Glessgen, C. Grab, D. Hits, W. Lustermann, A.-M. Lyon, R. A. Manzoni, L. Marchese, C. Martin Perez, A. Mascellani, F. Nessi-Tedaldi, F. Pauss, V. Perovic, S. Pigazzini, M. G. Ratti, M. Reichmann, C. Reissel, T. Reitenspiess, B. Ristic, F. Riti, D. Ruini, D. A. Sanz Becerra, R. Seidita, J. Steggemann, D. Valsecchi, R. Wallny, C. Amsler, P. Bärtschi, C. Botta, D. Brzhechko, M. F. Canelli, K. Cormier, A. De Wit, R. Del Burgo, J. K. Heikkilä, M. Huwiler, W. Jin, A. Jofrehei, B. Kilminster, S. Leontsinis, S. P. Liechti, A. Macchiolo, P. Meiring, V. M. Mikuni, U. Molinatti, I. Neutelings, A. Reimers, P. Robmann, S. Sanchez Cruz, K. Schweiger, M. Senger, Y. Takahashi, C. Adloff, C. M. Kuo, W. Lin, P. K. Rout, P. C. Tiwari, S. S. Yu, L. Ceard, Y. Chao, K. F. Chen, P. s. Chen, W.-S. Hou, Y. w. Kao, R. Khurana, G. Kole, Y. Y. Li, R.-S. Lu, E. Paganis, A. Psallidas, J. Thomas-Wilsker, H. y. Wu, E. Yazgan, C. Asawatangtrakuldee, N. Srimanobhas, V. Wachirapusitanand, D. Agyel, F. Boran, Z. S. Demiroglu, F. Dolek, I. Dumanoglu, E. Eskut, Y. Guler, E. Gurpinar Guler, C. Isik, O. Kara, A. Kayis Topaksu, U. Kiminsu, G. Onengut, K. Ozdemir, A. Polatoz, B. Tali, U. G. Tok, S. Turkcapar, E. Uslan, I. S. Zorbakir, K. Ocalan, M. Yalvac, B. Akgun, I. O. Atakisi, E. Gülmez, M. Kaya, O. Kaya, S. Tekten, A. Cakir, K. Cankocak, Y. Komurcu, S. Sen, O. Aydilek, S. Cerci, V. Epshteyn, B. Hacisahinoglu, I. Hos, B. Isildak, B. Kaynak, S. Ozkorucuklu, H. Sert, C. Simsek, D. Sunar Cerci, C. Zorbilmez, A. Boyaryntsev, B. Grynyov, L. Levchuk, D. Anthony, J. J. Brooke, A. Bundock, E. Clement, D. Cussans, H. Flacher, M. Glowacki, J. Goldstein, H. F. Heath, L. Kreczko, B. Krikler, S. Paramesvaran, S. Seif El Nasr-Storey, V. J. Smith, N. Stylianou, K. Walkingshaw Pass, R. White, A. H. Ball, K. W. Bell, A. Belyaev, C. Brew, R. M. Brown, D. J. A. Cockerill, C. Cooke, K. V. Ellis, K. Harder, S. Harper, M.-L. Holmberg, Sh. Jain, J. Linacre, K. Manolopoulos, D. M. Newbold, E. Olaiya, D. Petyt, T. Reis, G. Salvi, T. Schuh, C. H. Shepherd-Themistocleous, I. R. Tomalin, T. Williams, R. Bainbridge, P. Bloch, C. E. Brown, O. Buchmuller, V. Cacchio, C. A. Carrillo Montoya, V. Cepaitis, G. S. Chahal, D. Colling, J. S. Dancu, P. Dauncey, G. Davies, J. Davies, M. Della Negra, S. Fayer, G. Fedi, G. Hall, M. H. Hassanshahi, A. Howard, G. Iles, J. Langford, L. Lyons, A.-M. Magnan, S. Malik, A. Martelli, M. Mieskolainen, J. Nash, M. Pesaresi, B. C. Radburn-Smith, A. Richards, A. Rose, C. Seez, R. Shukla, A. Tapper, K. Uchida, G. P. Uttley, L. H. Vage, T. Virdee, M. Vojinovic, N. Wardle, D. Winterbottom, K. Coldham, J. E. Cole, A. Khan, P. Kyberd, I. D. Reid, S. Abdullin, A. Brinkerhoff, B. Caraway, J. Dittmann, K. Hatakeyama, J. Hiltbrand, A. R. Kanuganti, B. McMaster, M. Saunders, S. Sawant, C. Sutantawibul, M. Toms, J. Wilson, R. Bartek, A. Dominguez, C. Huerta Escamilla, A. E. Simsek, R. Uniyal, A. M. Vargas Hernandez, R. Chudasama, S. I. Cooper, S. V. Gleyzer, C. U. Perez, P. Rumerio, E. Usai, C. West, A. Akpinar, A. Albert, D. Arcaro, C. Cosby, Z. Demiragli, C. Erice, E. Fontanesi, D. Gastler, J. Rohlf, K. Salyer, D. Sperka, D. Spitzbart, I. Suarez, A. Tsatsos, S. Yuan, G. Benelli, X. Coubez, D. Cutts, M. Hadley, U. Heintz, J. M. Hogan, T. Kwon, G. Landsberg, K. T. Lau, D. Li, J. Luo, M. Narain, N. Pervan, S. Sagir, F. Simpson, W. Y. Wong, X. Yan, D. Yu, W. Zhang, S. Abbott, J. Bonilla, C. Brainerd, R. Breedon, M. Calderon De La Barca Sanchez, M. Chertok, J. Conway, P. T. Cox, R. Erbacher, G. Haza, F. Jensen, O. Kukral, G. Mocellin, M. Mulhearn, D. Pellett, B. Regnery, W. Wei, Y. Yao, F. Zhang, M. Bachtis, R. Cousins, A. Datta, J. Hauser, M. Ignatenko, M. A. Iqbal, T. Lam, E. Manca, W. A. Nash, D. Saltzberg, B. Stone, V. Valuev, R. Clare, M. Gordon, G. Hanson, W. Si, S. Wimpenny, J. G. Branson, S. Cittolin, S. Cooperstein, D. Diaz, J. Duarte, R. Gerosa, L. Giannini, J. Guiang, R. Kansal, V. Krutelyov, R. Lee, J. Letts, M. Masciovecchio, F. Mokhtar, M. Pieri, M. Quinnan, B. V. Sathia Narayanan, V. Sharma, M. Tadel, E. Vourliotis, F. Würthwein, Y. Xiang, A. Yagil, L. Brennan, C. Campagnari, M. Citron, G. Collura, A. Dorsett, J. Incandela, M. Kilpatrick, J. Kim, A. J. Li, P. Masterson, H. Mei, M. Oshiro, J. Richman, U. Sarica, R. Schmitz, F. Setti, J. Sheplock, D. Stuart, S. Wang, A. Bornheim, O. Cerri, A. Latorre, J. M. Lawhorn, J. Mao, H. B. Newman, T. Q. Nguyen, M. Spiropulu, J. R. Vlimant, C. Wang, S. Xie, R. Y. Zhu, J. Alison, S. An, M. B. Andrews, P. Bryant, V. Dutta, T. Ferguson, A. Harilal, C. Liu, T. Mudholkar, S. Murthy, M. Paulini, A. Roberts, A. Sanchez, W. Terrill, J. P. Cumalat, W. T. Ford, A. Hassani, G. Karathanasis, E. MacDonald, N. Manganelli, F. Marini, A. Perloff, C. Savard, N. Schonbeck, K. Stenson, K. A. Ulmer, S. R. Wagner, N. Zipper, J. Alexander, S. Bright-Thonney, X. Chen, D. J. Cranshaw, J. Fan, X. Fan, D. Gadkari, S. Hogan, J. Monroy, J. R. Patterson, J. Reichert, M. Reid, A. Ryd, J. Thom, P. Wittich, R. Zou, M. Albrow, M. Alyari, O. Amram, G. Apollinari, A. Apresyan, L. A. T. Bauerdick, D. Berry, J. Berryhill, P. C. Bhat, K. Burkett, J. N. Butler, A. Canepa, G. B. Cerati, H. W. K. Cheung, F. Chlebana, G. Cummings, J. Dickinson, I. Dutta, V. D. Elvira, Y. Feng, J. Freeman, A. Gandrakota, Z. Gecse, L. Gray, D. Green, S. Grünendahl, D. Guerrero, O. Gutsche, R. M. Harris, R. Heller, T. C. Herwig, J. Hirschauer, L. Horyn, B. Jayatilaka, S. Jindariani, M. Johnson, U. Joshi, T. Klijnsma, B. Klima, K. H. M. Kwok, S. Lammel, D. Lincoln, R. Lipton, T. Liu, C. Madrid, K. Maeshima, C. Mantilla, D. Mason, P. McBride, P. Merkel, S. Mrenna, S. Nahn, J. Ngadiuba, D. Noonan, V. Papadimitriou, N. Pastika, K. Pedro, C. Pena, F. Ravera, A. Reinsvold Hall, L. Ristori, E. Sexton-Kennedy, N. Smith, A. Soha, L. Spiegel, J. Strait, L. Taylor, S. Tkaczyk, N. V. Tran, L. Uplegger, E. W. Vaandering, I. Zoi, P. Avery, D. Bourilkov, L. Cadamuro, P. Chang, V. Cherepanov, R. D. Field, E. Koenig, M. Kolosova, J. Konigsberg, A. Korytov, K. H. Lo, K. Matchev, N. Menendez, G. Mitselmakher, A. Muthirakalayil Madhu, N. Rawal, D. Rosenzweig, S. Rosenzweig, K. Shi, J. Wang, T. Adams, A. Al Kadhim, A. Askew, N. Bower, R. Habibullah, V. Hagopian, R. Hashmi, R. S. Kim, S. Kim, T. Kolberg, G. Martinez, H. Prosper, P. R. Prova, O. Viazlo, M. Wulansatiti, R. Yohay, J. Zhang, B. Alsufyani, M. M. Baarmand, S. Butalla, T. Elkafrawy, M. Hohlmann, R. Kumar Verma, M. Rahmani, F. Yumiceva, M. R. Adams, C. Bennett, R. Cavanaugh, S. Dittmer, O. Evdokimov, C. E. Gerber, D. J. Hofman, J. H. Lee, D. S. Lemos, A. H. Merrit, C. Mills, S. Nanda, G. Oh, D. Pilipovic, T. Roy, S. Rudrabhatla, M. B. Tonjes, N. Varelas, X. Wang, Z. Ye, J. Yoo, M. Alhusseini, D. Blend, K. Dilsiz, L. Emediato, G. Karaman, O. K. Köseyan, J.-P. Merlo, A. Mestvirishvili, J. Nachtman, O. Neogi, H. Ogul, Y. Onel, A. Penzo, C. Snyder, E. Tiras, B. Blumenfeld, L. Corcodilos, J. Davis, A. V. Gritsan, L. Kang, S. Kyriacou, P. Maksimovic, M. Roguljic, J. Roskes, S. Sekhar, M. Swartz, T. Á. Vámi, A. Abreu, L. F. Alcerro Alcerro, J. Anguiano, P. Baringer, A. Bean, Z. Flowers, J. King, G. Krintiras, M. Lazarovits, C. Le Mahieu, C. Lindsey, J. Marquez, N. Minafra, M. Murray, M. Nickel, M. Pitt, S. Popescu, C. Rogan, C. Royon, R. Salvatico, S. Sanders, C. Smith, Q. Wang, G. Wilson, B. Allmond, S. Duric, A. Ivanov, K. Kaadze, A. Kalogeropoulos, D. Kim, Y. Maravin, T. Mitchell, K. Nam, J. Natoli, D. Roy, F. Rebassoo, D. Wright, E. Adams, A. Baden, O. Baron, A. Belloni, A. Bethani, Y. M. Chen, S. C. Eno, N. J. Hadley, S. Jabeen, R. G. Kellogg, T. Koeth, Y. Lai, S. Lascio, A. C. Mignerey, S. Nabili, C. Palmer, C. Papageorgakis, L. Wang, K. Wong, J. Bendavid, W. Busza, I. A. Cali, Y. Chen, M. D’Alfonso, J. Eysermans, C. Freer, G. Gomez-Ceballos, M. Goncharov, P. Harris, D. Hoang, D. Kovalskyi, J. Krupa, L. Lavezzo, Y.-J. Lee, K. Long, C. Mironov, C. Paus, D. Rankin, C. Roland, G. Roland, S. Rothman, Z. Shi, G. S. F. Stephans, J. Wang, Z. Wang, B. Wyslouch, T. J. Yang, R. M. Chatterjee, B. Crossman, B. M. Joshi, C. Kapsiak, M. Krohn, D. Mahon, J. Mans, M. Revering, R. Rusack, R. Saradhy, N. Schroeder, N. Strobbe, M. A. Wadud, L. M. Cremaldi, K. Bloom, M. Bryson, D. R. Claes, C. Fangmeier, F. Golf, C. Joo, I. Kravchenko, I. Reed, J. E. Siado, G. R. Snow, W. Tabb, A. Wightman, F. Yan, A. G. Zecchinelli, G. Agarwal, H. Bandyopadhyay, L. Hay, I. Iashvili, A. Kharchilava, C. McLean, M. Morris, D. Nguyen, J. Pekkanen, S. Rappoccio, H. Rejeb Sfar, A. Williams, G. Alverson, E. Barberis, Y. Haddad, Y. Han, A. Krishna, J. Li, G. Madigan, B. Marzocchi, D. M. Morse, V. Nguyen, T. Orimoto, A. Parker, L. Skinnari, A. Tishelman-Charny, B. Wang, D. Wood, S. Bhattacharya, J. Bueghly, Z. Chen, A. Gilbert, K. A. Hahn, Y. Liu, D. G. Monk, M. H. Schmitt, A. Taliercio, M. Velasco, R. Band, R. Bucci, M. Cremonesi, A. Das, R. Goldouzian, M. Hildreth, K. Hurtado Anampa, C. Jessop, K. Lannon, J. Lawrence, N. Loukas, L. Lutton, J. Mariano, N. Marinelli, I. Mcalister, T. McCauley, C. Mcgrady, K. Mohrman, C. Moore, Y. Musienko, H. Nelson, R. Ruchti, A. Townsend, M. Wayne, H. Yockey, M. Zarucki, L. Zygala, B. Bylsma, M. Carrigan, L. S. Durkin, C. Hill, M. Joyce, A. Lesauvage, M. Nunez Ornelas, K. Wei, B. L. Winer, B. R. Yates, F. M. Addesa, H. Bouchamaoui, P. Das, G. Dezoort, P. Elmer, A. Frankenthal, B. Greenberg, N. Haubrich, S. Higginbotham, G. Kopp, S. Kwan, D. Lange, A. Loeliger, D. Marlow, I. Ojalvo, J. Olsen, D. Stickland, C. Tully, S. Malik, A. S. Bakshi, V. E. Barnes, S. Chandra, R. Chawla, S. Das, A. Gu, L. Gutay, M. Jones, A. W. Jung, D. Kondratyev, A. M. Koshy, M. Liu, G. Negro, N. Neumeister, G. Paspalaki, S. Piperov, A. Purohit, J. F. Schulte, M. Stojanovic, J. Thieman, F. Wang, W. Xie, J. Dolen, N. Parashar, A. Pathak, D. Acosta, A. Baty, T. Carnahan, S. Dildick, K. M. Ecklund, P. J. Fernández Manteca, S. Freed, P. Gardner, F. J. M. Geurts, A. Kumar, W. Li, O. Miguel Colin, B. P. Padley, R. Redjimi, J. Rotter, S. Yang, E. Yigitbasi, Y. Zhang, A. Bodek, P. de Barbaro, R. Demina, J. L. Dulemba, C. Fallon, A. Garcia-Bellido, O. Hindrichs, A. Khukhunaishvili, P. Parygin, E. Popova, R. Taus, G. P. Van Onsem, K. Goulianos, B. Chiarito, J. P. Chou, Y. Gershtein, E. Halkiadakis, A. Hart, M. Heindl, D. Jaroslawski, O. Karacheban, I. Laflotte, A. Lath, R. Montalvo, K. Nash, M. Osherson, H. Routray, S. Salur, S. Schnetzer, S. Somalwar, R. Stone, S. A. Thayil, S. Thomas, J. Vora, H. Wang, H. Acharya, A. G. Delannoy, S. Fiorendi, T. Holmes, N. Karunarathna, L. Lee, E. Nibigira, S. Spanier, M. Ahmad, O. Bouhali, M. Dalchenko, A. Delgado, R. Eusebi, J. Gilmore, T. Huang, T. Kamon, H. Kim, S. Luo, S. Malhotra, R. Mueller, D. Overton, D. Rathjens, A. Safonov, N. Akchurin, J. Damgov, V. Hegde, A. Hussain, Y. Kazhykarim, K. Lamichhane, S. W. Lee, A. Mankel, T. Mengke, S. Muthumuni, T. Peltola, I. Volobouev, A. Whitbeck, E. Appelt, S. Greene, A. Gurrola, W. Johns, R. Kunnawalkam Elayavalli, A. Melo, F. Romeo, P. Sheldon, S. Tuo, J. Velkovska, J. Viinikainen, B. Cardwell, B. Cox, J. Hakala, R. Hirosky, A. Ledovskoy, A. Li, C. Neu, C. E. Perez Lara, P. E. Karchin, A. Aravind, S. Banerjee, K. Black, T. Bose, S. Dasu, I. De Bruyn, P. Everaerts, C. Galloni, H. He, M. Herndon, A. Herve, C. K. Koraka, A. Lanaro, R. Loveless, J. Madhusudanan Sreekala, A. Mallampalli, A. Mohammadi, S. Mondal, G. Parida, D. Pinna, A. Savin, V. Shang, V. Sharma, W. H. Smith, D. Teague, H. F. Tsoi, W. Vetens, A. Warden, S. Afanasiev, V. Andreev, Yu. Andreev, T. Aushev, M. Azarkin, A. Babaev, A. Belyaev, V. Blinov, E. Boos, V. Borshch, D. Budkouski, M. Chadeeva, V. Chekhovsky, A. Dermenev, T. Dimova, D. Druzhkin, M. Dubinin, L. Dudko, G. Gavrilov, V. Gavrilov, S. Gninenko, V. Golovtcov, N. Golubev, I. Golutvin, I. Gorbunov, A. Gribushin, Y. Ivanov, V. Kachanov, L. Kardapoltsev, V. Karjavine, A. Karneyeu, V. Kim, M. Kirakosyan, D. Kirpichnikov, M. Kirsanov, V. Klyukhin, O. Kodolova, D. Konstantinov, V. Korenkov, A. Kozyrev, N. Krasnikov, A. Lanev, P. Levchenko, O. Lukina, N. Lychkovskaya, V. Makarenko, A. Malakhov, V. Matveev, V. Murzin, A. Nikitenko, S. Obraztsov, V. Oreshkin, A. Oskin, V. Palichik, V. Perelygin, S. Petrushanko, S. Polikarpov, V. Popov, O. Radchenko, V. Rusinov, M. Savina, V. Savrin, D. Selivanova, V. Shalaev, S. Shmatov, S. Shulha, Y. Skovpen, S. Slabospitskii, V. Smirnov, A. Snigirev, D. Sosnov, V. Sulimov, E. Tcherniaev, A. Terkulov, O. Teryaev, I. Tlisova, A. Toropin, L. Uvarov, A. Uzunian, A. Vorobyev, N. Voytishin, B. S. Yuldashev, A. Zarubin, I. Zhizhin, A. Zhokin

**Affiliations:** 1 Yerevan Physics Institute, Yerevan, Armenia; 2 Institut für Hochenergiephysik, Vienna, Austria; 3 Universiteit Antwerpen, Antwerpen, Belgium; 4 Vrije Universiteit Brussel, Brussel, Belgium; 5 Université Libre de Bruxelles, Bruxelles, Belgium; 6 Ghent University, Ghent, Belgium; 7 Université Catholique de Louvain, Louvain-la-Neuve, Belgium; 8 Centro Brasileiro de Pesquisas Fisicas, Rio de Janeiro, Brazil; 9 Universidade do Estado do Rio de Janeiro, Rio de Janeiro, Brazil; 10 Universidade Estadual Paulista, Universidade Federal do ABC, São Paulo, Brazil; 11 Institute for Nuclear Research and Nuclear Energy, Bulgarian Academy of Sciences, Sofia, Bulgaria; 12 University of Sofia, Sofia, Bulgaria; 13 Instituto De Alta Investigación, Universidad de Tarapacá, Casilla 7 D, Arica, Chile; 14 Beihang University, Beijing, China; 15 Department of Physics, Tsinghua University, Beijing, China; 16 Institute of High Energy Physics, Beijing, China; 17 State Key Laboratory of Nuclear Physics and Technology, Peking University, Beijing, China; 18 Sun Yat-Sen University, Guangzhou, China; 19 University of Science and Technology of China, Hefei, China; 20 Institute of Modern Physics and Key Laboratory of Nuclear Physics and Ion-beam Application (MOE)-Fudan University, Shanghai, China; 21 Zhejiang University, Hangzhou, Zhejiang China; 22 Universidad de Los Andes, Bogota, Colombia; 23 Universidad de Antioquia, Medellin, Colombia; 24 Faculty of Electrical Engineering, Mechanical Engineering and Naval Architecture, University of Split, Split, Croatia; 25 Faculty of Science, University of Split, Split, Croatia; 26 Institute Rudjer Boskovic, Zagreb, Croatia; 27 University of Cyprus, Nicosia, Cyprus; 28 Charles University, Prague, Czech Republic; 29 Escuela Politecnica Nacional, Quito, Ecuador; 30 Universidad San Francisco de Quito, Quito, Ecuador; 31 Academy of Scientific Research and Technology of the Arab Republic of Egypt, Egyptian Network of High Energy Physics, Cairo, Egypt; 32 Center for High Energy Physics (CHEP-FU), Fayoum University, El-Fayoum, Egypt; 33 National Institute of Chemical Physics and Biophysics, Tallinn, Estonia; 34 Department of Physics, University of Helsinki, Helsinki, Finland; 35 Helsinki Institute of Physics, Helsinki, Finland; 36 Lappeenranta-Lahti University of Technology, Lappeenranta, Finland; 37 IRFU, CEA, Université Paris-Saclay, Gif-sur-Yvette, France; 38 Laboratoire Leprince-Ringuet, CNRS/IN2P3, Ecole Polytechnique, Institut Polytechnique de Paris, Palaiseau, France; 39 Université de Strasbourg, CNRS, IPHC UMR 7178, Strasbourg, France; 40 Institut de Physique des 2 Infinis de Lyon (IP2I ), Villeurbanne, France; 41 Georgian Technical University, Tbilisi, Georgia; 42 I. Physikalisches Institut, RWTH Aachen University, Aachen, Germany; 43 III. Physikalisches Institut A, RWTH Aachen University, Aachen, Germany; 44 III. Physikalisches Institut B, RWTH Aachen University, Aachen, Germany; 45 Deutsches Elektronen-Synchrotron, Hamburg, Germany; 46 University of Hamburg, Hamburg, Germany; 47 Karlsruher Institut fuer Technologie, Karlsruhe, Germany; 48 Institute of Nuclear and Particle Physics (INPP), NCSR Demokritos, Aghia Paraskevi, Greece; 49 National and Kapodistrian University of Athens, Athens, Greece; 50 National Technical University of Athens, Athens, Greece; 51 University of Ioánnina, Ioánnina, Greece; 52 HUN-REN Wigner Research Centre for Physics, Budapest, Hungary; 53 MTA-ELTE Lendület CMS Particle and Nuclear Physics Group, Eötvös Loránd University, Budapest, Hungary; 54 Institute of Nuclear Research ATOMKI, Debrecen, Hungary; 55 Institute of Physics, University of Debrecen, Debrecen, Hungary; 56 Karoly Robert Campus, MATE Institute of Technology, Gyongyos, Hungary; 57 Panjab University, Chandigarh, India; 58 University of Delhi, Delhi, India; 59 Saha Institute of Nuclear Physics, HBNI, Kolkata, India; 60 Indian Institute of Technology Madras, Madras, India; 61 Tata Institute of Fundamental Research-A, Mumbai, India; 62 Tata Institute of Fundamental Research-B, Mumbai, India; 63 National Institute of Science Education and Research, An OCC of Homi Bhabha National Institute, Bhubaneswar, Odisha India; 64 Indian Institute of Science Education and Research (IISER), Pune, India; 65 Isfahan University of Technology, Isfahan, Iran; 66 Institute for Research in Fundamental Sciences (IPM), Tehran, Iran; 67 University College Dublin, Dublin, Ireland; 68 INFN Sezione di Bari, Università di Bari, Politecnico di Bari, Bari, Italy; 69 INFN Sezione di Bologna, Università di Bologna, Bologna, Italy; 70 INFN Sezione di Catania, Università di Catania, Catania, Italy; 71 INFN Sezione di Firenze, Università di Firenze, Firenze, Italy; 72 INFN Laboratori Nazionali di Frascati, Frascati, Italy; 73 INFN Sezione di Genova, Università di Genova, Genoa, Italy; 74 INFN Sezione di Milano-Bicocca, Università di Milano-Bicocca, Milan, Italy; 75 INFN Sezione di Napoli, Università di Napoli ‘Federico II’, Napoli, Italy; Università della Basilicata, Potenza, Italy; Scuola Superiore Meridionale (SSM), Naples, Italy; 76 INFN Sezione di Padova, Università di Padova, Padova, Italy; Università di Trento, Trento, Italy; 77 INFN Sezione di Pavia, Università di Pavia, Pavia, Italy; 78 INFN Sezione di Perugia, Università di Perugia, Perugia, Italy; 79 INFN Sezione di Pisa, Università di Pisa, Scuola Normale Superiore di Pisa, Pisa, Italy; Università di Siena, Siena, Italy; 80 INFN Sezione di Roma, Sapienza Università di Roma, Rome, Italy; 81 INFN Sezione di Torino, Università di Torino, Torino, Italy; Università del Piemonte Orientale, Novara, Italy; 82 INFN Sezione di Trieste, Università di Trieste, Trieste, Italy; 83 Kyungpook National University, Daegu, Korea; 84 Chonnam National University, Institute for Universe and Elementary Particles, Kwangju, Korea; 85 Hanyang University, Seoul, Korea; 86 Korea University, Seoul, Korea; 87 Kyung Hee University, Department of Physics, Seoul, Korea; 88 Sejong University, Seoul, Korea; 89 Seoul National University, Seoul, Korea; 90 University of Seoul, Seoul, Korea; 91 Department of Physics, Yonsei University, Seoul, Korea; 92 Sungkyunkwan University, Suwon, Korea; 93 College of Engineering and Technology, American University of the Middle East (AUM), Dasman, Kuwait; 94 Riga Technical University, Riga, Latvia; 95 University of Latvia (LU), Riga, Latvia; 96 Vilnius University, Vilnius, Lithuania; 97 National Centre for Particle Physics, Universiti Malaya, Kuala Lumpur, Malaysia; 98 Universidad de Sonora (UNISON), Hermosillo, Mexico; 99 Centro de Investigacion y de Estudios Avanzados del IPN, Mexico City, Mexico; 100 Universidad Iberoamericana, Mexico City, Mexico; 101 Benemerita Universidad Autonoma de Puebla, Puebla, Mexico; 102 University of Montenegro, Podgorica, Montenegro; 103 University of Canterbury, Christchurch, New Zealand; 104 National Centre for Physics, Quaid-I-Azam University, Islamabad, Pakistan; 105 AGH University of Krakow, Faculty of Computer Science, Electronics and Telecommunications, Kraków, Poland; 106 National Centre for Nuclear Research, Swierk, Poland; 107 Institute of Experimental Physics, Faculty of Physics, University of Warsaw, Warsaw, Poland; 108 Warsaw University of Technology, Warsaw, Poland; 109 Laboratório de Instrumentação e Física Experimental de Partículas, Lisbon, Portugal; 110 Faculty of Physics, University of Belgrade, Belgrade, Serbia; 111 VINCA Institute of Nuclear Sciences, University of Belgrade, Belgrade, Serbia; 112 Centro de Investigaciones Energéticas Medioambientales y Tecnológicas (CIEMAT), Madrid, Spain; 113 Universidad Autónoma de Madrid, Madrid, Spain; 114 Universidad de Oviedo, Instituto Universitario de Ciencias y Tecnologías Espaciales de Asturias (ICTEA), Oviedo, Spain; 115 Instituto de Física de Cantabria (IFCA), CSIC-Universidad de Cantabria, Santander, Spain; 116 University of Colombo, Colombo, Sri Lanka; 117 Department of Physics, University of Ruhuna, Matara, Sri Lanka; 118 CERN, European Organization for Nuclear Research, Geneva, Switzerland; 119 Paul Scherrer Institut, Villigen, Switzerland; 120 ETH Zurich-Institute for Particle Physics and Astrophysics (IPA), Zurich, Switzerland; 121 Universität Zürich, Zurich, Switzerland; 122 National Central University, Chung-Li, Taiwan; 123 National Taiwan University (NTU), Taipei, Taiwan; 124 High Energy Physics Research Unit, Department of Physics, Faculty of Science, Chulalongkorn University, Bangkok, Thailand; 125 Physics Department, Science and Art Faculty, Çukurova University, Adana, Turkey; 126 Physics Department, Middle East Technical University, Ankara, Turkey; 127 Bogazici University, Istanbul, Turkey; 128 Istanbul Technical University, Istanbul, Turkey; 129 Istanbul University, Istanbul, Turkey; 130 Institute for Scintillation Materials of National Academy of Science of Ukraine, Kharkiv, Ukraine; 131 National Science Centre, Kharkiv Institute of Physics and Technology, Kharkiv, Ukraine; 132 University of Bristol, Bristol, UK; 133 Rutherford Appleton Laboratory, Didcot, UK; 134 Imperial College, London, UK; 135 Brunel University, Uxbridge, UK; 136 Baylor University, Waco, TX USA; 137 Catholic University of America, Washington, DC USA; 138 The University of Alabama, Tuscaloosa, AL USA; 139 Boston University, Boston, MA USA; 140 Brown University, Providence, RI USA; 141 University of California, Davis, Davis, CA USA; 142 University of California, Los Angeles, CA USA; 143 University of California, Riverside, Riverside, CA USA; 144 University of California, San Diego, La Jolla, CA USA; 145 Department of Physics, University of California, Santa Barbara, Santa Barbara, CA USA; 146 California Institute of Technology, Pasadena, CA USA; 147 Carnegie Mellon University, Pittsburgh, PA USA; 148 University of Colorado Boulder, Boulder, CO USA; 149 Cornell University, Ithaca, NY USA; 150 Fermi National Accelerator Laboratory, Batavia, IL USA; 151 University of Florida, Gainesville, FL USA; 152 Florida State University, Tallahassee, FL USA; 153 Florida Institute of Technology, Melbourne, FL USA; 154 University of Illinois Chicago, Chicago, USA; 155 The University of Iowa, Iowa City, IA USA; 156 Johns Hopkins University, Baltimore, MD USA; 157 The University of Kansas, Lawrence, KS USA; 158 Kansas State University, Manhattan, KS USA; 159 Lawrence Livermore National Laboratory, Livermore, CA USA; 160 University of Maryland, College Park, MD USA; 161 Massachusetts Institute of Technology, Cambridge, MA USA; 162 University of Minnesota, Minneapolis, MN USA; 163 University of Mississippi, Oxford, MS USA; 164 University of Nebraska-Lincoln, Lincoln, NE USA; 165 State University of New York at Buffalo, Buffalo, NY USA; 166 Northeastern University, Boston, MA USA; 167 Northwestern University, Evanston, IL USA; 168 University of Notre Dame, Notre Dame, IN USA; 169 The Ohio State University, Columbus, OH USA; 170 Princeton University, Princeton, NJ USA; 171 University of Puerto Rico, Mayaguez, PR USA; 172 Purdue University, West Lafayette, IN USA; 173 Purdue University Northwest, Hammond, IN USA; 174 Rice University, Houston, TX USA; 175 University of Rochester, Rochester, NY USA; 176 The Rockefeller University, New York, NY USA; 177 Rutgers, The State University of New Jersey, Piscataway, NJ USA; 178 University of Tennessee, Knoxville, TN USA; 179 Texas A&M University, College Station, TX USA; 180 Texas Tech University, Lubbock, TX USA; 181 Vanderbilt University, Nashville, TN USA; 182 University of Virginia, Charlottesville, VA USA; 183 Wayne State University, Detroit, MI USA; 184 University of Wisconsin-Madison, Madison, WI USA; 185 Authors affiliated with an institute or an international laboratory covered by a cooperation agreement with CERN, Geneva, Switzerland; 186 Yerevan State University, Yerevan, Armenia; 187 TU Wien, Vienna, Austria; 188 Institute of Basic and Applied Sciences, Faculty of Engineering, Arab Academy for Science, Technology and Maritime Transport, Alexandria, Egypt; 189 Université Libre de Bruxelles, Bruxelles, Belgium; 190 Universidade Estadual de Campinas, Campinas, Brazil; 191 Federal University of Rio Grande do Sul, Porto Alegre, Brazil; 192 UFMS, Nova Andradina, Brazil; 193 University of Chinese Academy of Sciences, Beijing, China; 194 Nanjing Normal University, Nanjing, China; 195 Now at The University of Iowa, Iowa City, IA USA; 196 an institute or an international laboratory covered by a cooperation agreement with CERN, Geneva, Switzerland; 197 Helwan University, Cairo, Egypt; 198 Now at Zewail City of Science and Technology, Zewail, Egypt; 199 British University in Egypt, Cairo, Egypt; 200 Now at Ain Shams University, Cairo, Egypt; 201 Purdue University, West Lafayette, Indiana, USA; 202 Université de Haute Alsace, Mulhouse, France; 203 Department of Physics, Tsinghua University, Beijing, China; 204 The University of the State of Amazonas, Manaus, Brazil; 205 Erzincan Binali Yildirim University, Erzincan, Turkey; 206 University of Hamburg, Hamburg, Germany; 207 III. Physikalisches Institut A, RWTH Aachen University, Aachen, Germany; 208 Isfahan University of Technology, Isfahan, Iran; 209 Bergische University Wuppertal (BUW), Wuppertal, Germany; 210 Brandenburg University of Technology, Cottbus, Germany; 211 Forschungszentrum Jülich, Juelich, Germany; 212 CERN, European Organization for Nuclear Research, Geneva, Switzerland; 213 Institute of Physics, University of Debrecen, Debrecen, Hungary; 214 Institute of Nuclear Research ATOMKI, Debrecen, Hungary; 215 Now at Universitatea Babes-Bolyai-Facultatea de Fizica, Cluj-Napoca, Romania; 216 Physics Department, Faculty of Science, Assiut University, Assiut, Egypt; 217 HUN-REN Wigner Research Centre for Physics, Budapest, Hungary; 218 Faculty of Informatics, University of Debrecen, Debrecen, Hungary; 219 Punjab Agricultural University, Ludhiana, India; 220 UPES-University of Petroleum and Energy Studies, Dehradun, India; 221 University of Visva-Bharati, Santiniketan, India; 222 University of Hyderabad, Hyderabad, India; 223 Indian Institute of Science (IISc), Bangalore, India; 224 IIT Bhubaneswar, Bhubaneswar, India; 225 Institute of Physics, Bhubaneswar, India; 226 Deutsches Elektronen-Synchrotron, Hamburg, Germany; 227 Now at Department of Physics, Isfahan University of Technology, Isfahan, Iran; 228 Sharif University of Technology, Tehran, Iran; 229 Department of Physics, University of Science and Technology of Mazandaran, Behshahr, Iran; 230 Italian National Agency for New Technologies, Energy and Sustainable Economic Development, Bologna, Italy; 231 Centro Siciliano di Fisica Nucleare e di Struttura Della Materia, Catania, Italy; 232 Università degli Studi Guglielmo Marconi, Rome, Italy; 233 Scuola Superiore Meridionale, Università di Napoli ’Federico II’, Naples, Italy; 234 Fermi National Accelerator Laboratory, Batavia, IL USA; 235 Laboratori Nazionali di Legnaro dell’INFN, Legnaro, Italy; 236 Università di Napoli ’Federico II’, Naples, Italy; 237 Consiglio Nazionale delle Ricerche - Istituto Officina dei Materiali, Perugia, Italy; 238 IRFU, CEA, Université Paris-Saclay, Gif-sur-Yvette, France; 239 Riga Technical University, Riga, Latvia; 240 Department of Applied Physics, Faculty of Science and Technology, Universiti Kebangsaan Malaysia, Bangi, Malaysia; 241 Consejo Nacional de Ciencia y Tecnología, Mexico City, Mexico; 242 Trincomalee Campus, Eastern University, Nilaveli, Sri Lanka; 243 INFN Sezione di Pavia, Università di Pavia, Pavia, Italy; 244 National and Kapodistrian University of Athens, Athens, Greece; 245 Ecole Polytechnique Fédérale Lausanne, Lausanne, Switzerland; 246 Universität Zürich, Zurich, Switzerland; 247 Stefan Meyer Institute for Subatomic Physics, Vienna, Austria; 248 Laboratoire d’Annecy-le-Vieux de Physique des Particules, IN2P3-CNRS, Annecy-le-Vieux, France; 249 Near East University, Research Center of Experimental Health Science, Mersin, Turkey; 250 Konya Technical University, Konya, Turkey; 251 Izmir Bakircay University, Izmir, Turkey; 252 Adiyaman University, Adiyaman, Turkey; 253 Necmettin Erbakan University, Konya, Turkey; 254 Bozok Universitetesi Rektörlügü, Yozgat, Turkey; 255 Marmara University, Istanbul, Turkey; 256 Milli Savunma University, Istanbul, Turkey; 257 Kafkas University, Kars, Turkey; 258 Hacettepe University, Ankara, Turkey; 259 Faculty of Engineering, Istanbul University-Cerrahpasa, Istanbul, Turkey; 260 Ozyegin University, Istanbul, Turkey; 261 Vrije Universiteit Brussel, Brussel, Belgium; 262 School of Physics and Astronomy, University of Southampton, Southampton, UK; 263 University of Bristol, Bristol, UK; 264 IPPP Durham University, Durham, UK; 265 Monash University, Faculty of Science, Clayton, Australia; 266 Università di Torino, Turin, Italy; 267 Bethel University, St. Paul, MN USA; 268 Karamanoğlu Mehmetbey University, Karaman, Turkey; 269 California Institute of Technology, Pasadena, CA USA; 270 United States Naval Academy, Annapolis, MD USA; 271 Bingol University, Bingol, Turkey; 272 Georgian Technical University, Tbilisi, Georgia; 273 Sinop University, Sinop, Turkey; 274 Erciyes University, Kayseri, Turkey; 275 Horia Hulubei National Institute of Physics and Nuclear Engineering (IFIN-HH), Bucharest, Romania; 276 Texas A&M University at Qatar, Doha, Qatar; 277 Kyungpook National University, Daegu, Korea; 278 another institute or international laboratory covered by a cooperation agreement with CERN, Geneva, Switzerland; 279 Universiteit Antwerpen, Antwerpen, Belgium; 280 Yerevan Physics Institute, Yerevan, Armenia; 281 Northeastern University, Boston, MA USA; 282 Now at another institute or international laboratory covered by a cooperation agreement with CERN, Geneva, Switzerland; 283 Imperial College, London, UK; 284 Institute of Nuclear Physics of the Uzbekistan Academy of Sciences, Tashkent, Uzbekistan; 285 CERN, 1211 Geneva 23, Switzerland

## Abstract

A measurement of the dijet production cross section is reported based on proton–proton collision data collected in 2016 at $$\sqrt{s}=13\,\text {Te}\hspace{-.08em}\text {V} $$ by the CMS experiment at the CERN LHC, corresponding to an integrated luminosity of up to 36.3$$\,\text {fb}^{-1}$$. Jets are reconstructed with the anti-$$k_{\textrm{T}} $$ algorithm for distance parameters of $$R=0.4$$ and 0.8. Cross sections are measured double-differentially (2D) as a function of the largest absolute rapidity $$|y |_{\text {max}} $$ of the two jets with the highest transverse momenta $$p_{\textrm{T}}$$ and their invariant mass $$m_{1,2} $$, and triple-differentially (3D) as a function of the rapidity separation $$y^{*} $$, the total boost $$y_{\text {b}} $$, and either $$m_{1,2} $$ or the average $$p_{\textrm{T}}$$ of the two jets. The cross sections are unfolded to correct for detector effects and are compared with fixed-order calculations derived at next-to-next-to-leading order in perturbative quantum chromodynamics. The impact of the measurements on the parton distribution functions and the strong coupling constant at the mass of the $${\text {Z}} $$ boson is investigated, yielding a value of $$\alpha _\textrm{S} (m_{{\text {Z}}}) =0.1179\pm 0.0019$$.

## Introduction

The production of jets in high-energy proton–proton (${\text {p}} {\text {p}}$) collisions provides an important experimental input for the determination of the proton structure in terms of parton distribution functions (PDFs), and for the study of the strong force described by quantum chromodynamics (QCD). In conjunction with deep-inelastic ${\text {e}} ^{\pm }{\text {p}}$ scattering (DIS) measurements [1, 2], which strongly constrain the quark PDFs, jet data from ${\text {p}} {\text {p}}$ collisions at the LHC provide sensitivity to the gluon content and allow the running of the strong coupling constant $\alpha _\textrm{S}$ to be probed over a wide range of momentum scales. Recent progress made in calculating predictions for these processes at next-to-next-to-leading order (NNLO) accuracy [3, 4] in perturbative QCD (pQCD) underscores the need for precise experimental data up to the highest accessible energies.

Dijet observables are particularly well-suited for this purpose owing to the abundant production of jets in hadron-induced processes across a large phase space, which makes it possible to perform high-precision multi-differential measurements. Such measurements performed at the LHC include a triple-differential (3D) dijet measurement at a center-of-mass energy $\sqrt{s}=8\,\text {Te}\hspace{-.08em}\text {V}$ [5] using jets reconstructed with the anti-$k_{\textrm{T}}$ clustering algorithm [6, 7] with a distance parameter $R = 0.7$, and several double-differential (2D) measurements at 7 and 13$\,\text {Te}\hspace{-.08em}\text {V}$  [8–12] for anti-$k_{\textrm{T}}$ jets with $R = 0.4$, 0.6, or 0.7.

In this article, measurements of the dijet production cross section in ${\text {p}} {\text {p}}$ collisions at $\sqrt{s} = 13\,\text {Te}\hspace{-.08em}\text {V}$ from the CMS Collaboration are presented, using anti-$k_{\textrm{T}}$ jets for two values of the distance parameter, $R = 0.4$ and 0.8. Both 2D and 3D measurements are performed as a function of the kinematic properties of the two jets with the highest transverse momenta ($p_{\textrm{T}}$) in the event.

In the 2D case, the cross section is measured as a function of the largest absolute rapidity $$|y |_{\text {max}} $$ of the two jets and the invariant mass $$m_{1,2} $$ of the dijet system, as done for the CMS measurements at $$\sqrt{s} = 7\,\text {Te}\hspace{-.08em}\text {V} $$ [9, 10]. For the 3D measurements, the same two angular observables are considered as for the previous CMS measurement at $$\sqrt{s} = 8\,\text {Te}\hspace{-.08em}\text {V} $$ [5]: the dijet rapidity separation $$y^{*} =|y_1-y_2 |/2$$ and the total boost of the dijet system $$y_{\text {b}} =|y_1+y_2 |/2$$, where $$y_1$$ and $$y_2$$ indicate the rapidities of the jets. The measurements are performed as a function of $$y^{*} $$, $$y_{\text {b}} $$, and $$m_{1,2} $$, and alternatively as a function of $$y^{*} $$, $$y_{\text {b}} $$, and the average $$p_{\textrm{T}}$$ of the two jets, $$\langle p_{\textrm{T}} \rangle _{1,2} $$.

The 2D and 3D measurements cover a largely overlapping phase space. However, each of the two presents different experimental advantages stemming from the difference in the information content of the respective observables. The 2D measurement features a more inclusive rapidity binning, leading to an increased statistical precision and a larger accessible range in $$m_{1,2} $$. The use of two angular observables for the 3D measurement provides additional information on the dijet topology, at the expense of a reduced reach in $$m_{1,2} $$. Moreover, the variables $$y^{*} $$ and $$y_{\text {b}} $$ encode the dependence on the partonic scattering angle in the laboratory frame and the imbalance in the initial-state parton momenta, respectively. This is advantageous for comparisons to fixed-order pQCD predictions, which are obtained by convolving the partonic scattering cross sections and the PDFs. Specifically, the relation between the dijet invariant mass and the proton momentum fractions $$x_{\pm }$$ carried by the incoming partons is given at leading order (LO) by $$x_{\pm } = m_{1,2} ~\exp (\pm y_{\text {b}}) / \sqrt{s}$$. Using the average dijet $$p_{\textrm{T}}$$ instead renders the $$y^{*} $$ dependence explicit and gives $$x_{\pm } = 2\langle p_{\textrm{T}} \rangle _{1,2} ~\text {cosh}(y^{*})\exp (\pm y_{\text {b}}) / \sqrt{s}$$.

This article is organized as follows. A brief description of the CMS detector is given in Sect. 2. Section 3 presents the samples of recorded and simulated events used for the measurement. In Sect. 4, the reconstruction of the event content is described, and the selection criteria applied to events entering this analysis are given. Sections 5 and 6 detail the measurement of the 2D and 3D dijet cross sections using the reconstructed jets, and the unfolding of the resulting spectra to correct for detector effects, respectively. The different sources of experimental uncertainty in the measurement are outlined in Sect. 7. The measurements are compared to fixed-order predictions obtained at NNLO accuracy in pQCD, which are discussed in Sect. 8. A comparison of the measurements to the predictions obtained for several global PDF sets is presented in Sect. 9. In Sect. 10, the impact of including the present measurements in determinations of PDFs and the strong coupling constant at the scale of the Z boson mass, $\alpha _\textrm{S} (m_{{\text {Z}}})$, is investigated. Finally, a summary of the main findings is given in Sect. 11.

Tabulated results are provided in the HEPData record for this analysis [13].

## The CMS detector

The central feature of the CMS apparatus is a superconducting solenoid of 6$\,\text {m}$ internal diameter, providing a magnetic field of 3.8$\,\text {T}$. Within the solenoid volume are a silicon pixel and strip tracker, a lead tungstate crystal electromagnetic calorimeter (ECAL), and a brass and scintillator hadron calorimeter (HCAL), each composed of a barrel and two endcap sections. Forward calorimeters extend the pseudorapidity ($\eta$) coverage provided by the barrel and endcap detectors. Muons are detected in gas-ionization chambers embedded in the steel flux-return yoke outside the solenoid.

Events of interest are selected using a two-tiered trigger system [14]. The first level, composed of custom hardware processors, uses information from the calorimeters and muon detectors to select events at a rate of around 100$\,\text {kHz}$ within a fixed latency of about 4$\upmu$ s [15]. The second level, known as the high-level trigger (HLT), consists of a processor farm running a compact version of the full event reconstruction software, optimized for fast processing, and reduces the event rate to around 1$\,\text {kHz}$ before data storage.

A more detailed description of the CMS detector, together with a definition of the coordinate system used and the relevant kinematic variables, can be found in Ref. [16].

## Data and simulated samples

The measurements presented in this article are based on ${\text {p}} {\text {p}}$ collision data recorded by the CMS detector in 2016 at $\sqrt{s} = 13\,\text {Te}\hspace{-.08em}\text {V}$, corresponding to an integrated luminosity of up to 36.3$\,\text {fb}^{-1}$. Collision events containing jets are identified during data taking by dedicated trigger algorithms. Owing to the stringent timing constraints, jets at the HLT are clustered from particle candidates reconstructed using a simplified procedure, as compared to the full offline reconstruction.

The integrated luminosity recorded by the available jet-related triggers is given in Table 1. Several sets of triggers are deployed, which require the presence of at least one jet (two jets) with a $p_{\textrm{T}}$ (average $p_{\textrm{T}}$) above certain predefined thresholds. While distinct sets of single-jet triggers are deployed for anti-$k_{\textrm{T}}$ jets with distance parameters of $R = 0.4$ and 0.8, only the former are used for the dijet triggers. The integrated luminosity delivered by each of these triggers depends on the total time period during which it was deployed. In addition, low-threshold triggers are prescaled by a factor that is continually adjusted during data taking to optimize the data acquisition rate, resulting in lower effective integrated luminosities.

**Table 1:** Overview of the single-jet (dijet) triggers deployed for the different $p_{\textrm{T}}$ ($\langle p_{\textrm{T}} \rangle$) thresholds at the HLT, and the corresponding integrated luminosities

	Trigger threshold ($\text {Ge}\hspace{-.08em}\text {V}$ )				
	40	60	80	140	200
Trigger set	Int. luminosity ($\text {pb}^{-1}$ )				
Single-jet $R = 0.4$	0.3	0.7	2.8	24.2	103.6
Single-jet $R = 0.8$	0.05	0.3	1.0	10.1	85.8
Dijet $R = 0.4$	0.1	1.7	4.2	27.9	140.2

	Trigger threshold ($\text {Ge}\hspace{-.08em}\text {V}$ )				
	260	320	400	450	500
Trigger set	Int. luminosity ($\text {fb}^{-1}$ )				
Single-jet $R = 0.4$	0.6	1.8	5.2	36.3	36.3
Single-jet $R = 0.8$	0.5	1.5	4.6	33.5	33.5
Dijet $R = 0.4$	0.5	3.0	9.1	–	29.6

To study the impact of the detector response on the measurement, samples of simulated events produced using Monte Carlo (MC) event generators are used. Events are generated at LO in pQCD using pythia  8 [17] (version 212) with the CUETP8M1 tune [18]. The matrix element calculation is matched to the parton shower and takes multi-parton interactions and hadronization effects into account. An alternative LO sample, generated using the MadGraph 5_amc@nlo program [19] (version 2.2.2) and interfaced with pythia for the simulation of parton showering and hadronization, is used to estimate the dependence of results on the simulation model.

To simulate contributions from additional ${\text {p}} {\text {p}}$ collisions (pileup), the particles emerging from the high-energy scattering are overlaid with simulated minimum-bias events and propagated through a full simulation of the CMS detector modeled using the Geant4 package [20]. The resulting signals are then processed using the same reconstruction techniques used for collision data. Differences between the simulated and measured pileup activity are accounted for using a global reweighting of simulated events based on the mean number of pileup interactions determined in data based on an estimated inelastic ${\text {p}} {\text {p}}$ collision cross section of 69.2$\,\text {mb}$. This number is obtained using the pileup counting method described in the inelastic cross section measurement [21]. About 23 pileup interactions occurred for each proton bunch collision during the 2016 data taking [22].

## Event reconstruction and selection

A global description of collision events is achieved following the particle-flow approach [23], which aims to identify and measure the kinematic properties of each individual particle emerging from the collision using an optimized combination of information from the various elements of the CMS detector.

The trajectories of charged particles, as well as their originating ${\text {p}} {\text {p}}$ interaction vertices are reconstructed from hits in the inner tracking detectors. The primary vertex is taken to be the vertex corresponding to the hardest scattering in the event, evaluated using tracking information alone, as described in Section 9.4.1 of Ref. [24].

Muons are identified as particle tracks in the inner detector layers that are compatible with either a track or several hits in the muon system, and are associated with calorimeter deposits consistent with the muon hypothesis. The muon four-momentum is determined by fitting the muon trajectory using information from both the inner tracker and the muon system.

Photons are identified as ECAL energy clusters not linked to the extrapolation of any charged-particle track to the ECAL. Electrons are identified by linking a primary charged-particle track to potential energy deposits in the ECAL. The resulting energy clusters are required to be spatially compatible with the extrapolated track to the ECAL, or consistent with bremsstrahlung photons emitted in the tracker material. While for photons the energy is obtained directly from the ECAL measurement, the electron energy is determined from a combination of the track momentum at the primary interaction vertex and the associated ECAL clusters.

Charged hadrons are identified as particle tracks not identified as electrons or muons, and neutral hadrons are identified as HCAL energy clusters not linked to any charged-hadron trajectory, or as a combined ECAL and HCAL energy excess with respect to the expected charged hadron energy deposit. The energy of charged hadrons is determined by combining the track momentum and the corresponding ECAL and HCAL energies, corrected for the response function of the calorimeters to hadronic showers. The energy of neutral hadrons is obtained from the corresponding corrected ECAL and HCAL energies.

For each event, jets are clustered from the reconstructed particle candidates using the infrared- and collinear-safe anti-$k_{\textrm{T}}$ algorithm [6, 7] with distance parameters of $R = 0.4$ and 0.8. The jet momentum is determined as the vector sum of all particle momenta in the jet, and is found from simulation to be, on average, within 5–10% of the true momentum over the entire $p_{\textrm{T}}$ range and detector acceptance used in the analysis. To mitigate the effect of pileup, which can contribute additional tracks and calorimetric energy depositions to the jet momentum, charged particles identified as originating from pileup vertices are discarded and an offset correction [25] is applied to account for the remaining contributions.

Jet energy corrections [26] are derived from simulation studies so that the average energy of reconstructed jets becomes identical to that of particle-level jets. The latter are defined as jets clustered from all stable particles produced in the collision, excluding neutrinos. In situ measurements of the momentum balance in dijet, $\text {photon} + \text {jet}$, ${\text {Z}} + \text {jet}$, and multijet events are used to account for any residual differences in the jet energy scale (JES) between data and simulation. The jet energy resolution (JER) typically amounts to 15–20% at 30$\,\text {Ge}\hspace{-.08em}\text {V}$, 10% at 100$\,\text {Ge}\hspace{-.08em}\text {V}$, and 5% at 1$\,\text {Te}\hspace{-.08em}\text {V}$  [26]. It is measured in data using similar jet balancing approaches as for the JES, and residual differences between data and simulation are corrected by smearing the $p_{\textrm{T}}$ of simulated jets accordingly. Additional selection criteria [27] are applied to each jet to remove jets potentially dominated by spurious contributions from various subdetector components or reconstruction failures. Similarly, anomalous events caused by reconstruction failures, detector malfunctions, or noncollision backgrounds are identified and rejected by dedicated event filters. These are designed to identify more than 85–90% of anomalous events with a misidentification rate of less than 0.1%. Further details can be found in Ref. [28].

Events entering the 2D cross section measurements for both $R = 0.4$ and 0.8 are required to have been accepted by at least one single-jet trigger path operating on jets with the same distance parameter. For the 3D measurements, the dijet triggers are used on account of their lower overall prescale values.

To guarantee a high reconstruction efficiency at the trigger level, trigger paths with different thresholds are assigned to mutually exclusive phase space regions. These are determined for single-jet (dijet) triggers based on measurements of the trigger efficiency as a function of the leading jet $p_{\textrm{T}}$ (average $p_{\textrm{T}}$ of the two leading jets), requiring the trigger efficiency to remain above 99.5% in each region.

During the 2016 data taking, a gradual shift in the timing of the inputs of the ECAL first-level trigger in the region defined by $|\eta | > 2.0$ caused the trigger signal to be incorrectly associated to the previous bunch crossing (“prefiring”), leading to a specific trigger inefficiency. For events containing a jet with a $p_{\textrm{T}}$ larger than $\approx$100$\,\text {Ge}\hspace{-.08em}\text {V}$, the efficiency loss in the region $2.5< |\eta | < 3.0$ is $\approx$10–20%, depending on $p_{\textrm{T}}$, $\eta$, and time. Correction factors were computed from data and applied to the acceptance evaluated from simulation.

Further selection criteria are applied to events passing the trigger selection, based on the kinematic properties of two jets with the highest $p_{\textrm{T}}$, denoted in the following by the subscripts 1 and 2 for the $p_{\textrm{T}}$-leading and $p_{\textrm{T}}$-subleading jets, respectively. For the former, a requirement of $p_\text {T,1} >100\,\text {Ge}\hspace{-.08em}\text {V}$ and $|y_1 |<3$ is imposed, while the latter is required to satisfy $p_\text {T,2} >50\,\text {Ge}\hspace{-.08em}\text {V}$ and $|y_2 |<3$.

## Cross section measurement

The dijet production cross section is measured both double- and triple-differentially for anti-$$k_{\textrm{T}}$$ jets with distance parameters of $$R = 0.4$$ and 0.8 in terms of the properties of the system formed by the two $$p_{\textrm{T}}$$-leading jets. The 2D spectra are reconstructed as a function of $$m_{1,2} $$ in five rapidity regions defined in terms of the variable $$|y |_{\text {max}} = |y_{\text {max}} |$$, where $$y_{\text {max}} $$ corresponds to the rapidity of the jet closer to the beam line (outermost jet), and is given by

1 $$\begin{aligned} y_{\text {max}}= &   \text {sign}(|\text {max}(y_1, y_2) | - |\text {min}(y_1, y_2) |)\nonumber \\  &   \times \text {max}(|y_1 |, |y_2 |). \end{aligned}$$

In the 3D case, the cross section is measured as a function of $$m_{1,2} $$ and $$\langle p_{\textrm{T}} \rangle _{1,2} $$ in 15 rapidity regions, defined in terms of the dijet rapidity separation $$y^{*}$$ and the total boost $$y_{\text {b}}$$ of the dijet system, as given by

2 $$\begin{aligned} y^{*} = \frac{1}{2}|y_1 - y_2 |,\quad y_{\text {b}} = \frac{1}{2}|y_1 + y_2 |. \end{aligned}$$

The variables $$m_{1,2} $$ and $$\langle p_{\textrm{T}} \rangle _{1,2} $$ are obtained as

3 $$\begin{aligned} \begin{aligned} m_{1,2}&= \sqrt{(E_1 + E_2)^2 - (\vec {p}_1 + \vec {p}_2)^2},\\ \langle p_{\textrm{T}} \rangle _{1,2}&= \frac{1}{2}\left( p_\text {T,1} + p_\text {T,2} \right) , \end{aligned} \end{aligned}$$

where the subscripts 1 and 2 refer to the $p_{\textrm{T}}$-leading and $p_{\textrm{T}}$-subleading jets, respectively.

The differential dijet spectra are reconstructed from the effective event yield $N_{\text {eff}}$ in bins of the chosen observables, normalized to the integrated luminosity $\mathcal {L}_\text {int}$. The effective event yield is calculated from the raw event yield, taking into account the selection efficiency and subtracting background contributions. In addition, events that enter the measurement are weighted according to the prescale factor of the trigger path assigned to the corresponding phase space region.

The 2D cross section is obtained as a function of $$y_{\text {max}} $$ and $$m_{1,2} $$ as

4 $$\begin{aligned} \begin{aligned} \frac{\textrm{d}^2\sigma }{\textrm{d}y_{\text {max}}\textrm{d}m_{1,2}}&= \frac{1}{\mathcal {L}_\text {int}}\,\frac{N_{\text {eff}}}{(2\,\varDelta |y |_{\text {max}})\varDelta m_{1,2}}. \end{aligned} \end{aligned}$$

Here, $$\varDelta |y |_{\text {max}} $$ and $$\varDelta m_{1,2} $$ denote the width of the bins in the respective quantities. The measurement is performed in five rapidity bins of equal size within $$0< |y |_{\text {max}} < 2.5$$ and covers an invariant mass range of $$249< m_{1,2} < 10{,}050\,\text {Ge}\hspace{-.08em}\text {V} $$. The measurement boundaries are chosen starting from a preliminary binning determined using simulated samples based on the expected experimental resolution in $$m_{1,2} $$, and discarding bins at low $$m_{1,2} $$ that do not meet the minimal trigger efficiency requirement of 99.5%, and bins at high $$m_{1,2} $$ for which the statistical uncertainty exceeds 50%.

For the 3D measurement, the cross section is obtained as

5 $$\begin{aligned} \begin{aligned} \frac{\textrm{d}^3\sigma }{\textrm{d}y^{*} \textrm{d}y_{\text {b}} \textrm{d}x}&= \frac{1}{\mathcal {L}_\text {int}}\,\frac{N_{\text {eff}}}{\varDelta y^{*} \varDelta y_{\text {b}} \varDelta x}. \end{aligned} \end{aligned}$$

**Fig. 1:**
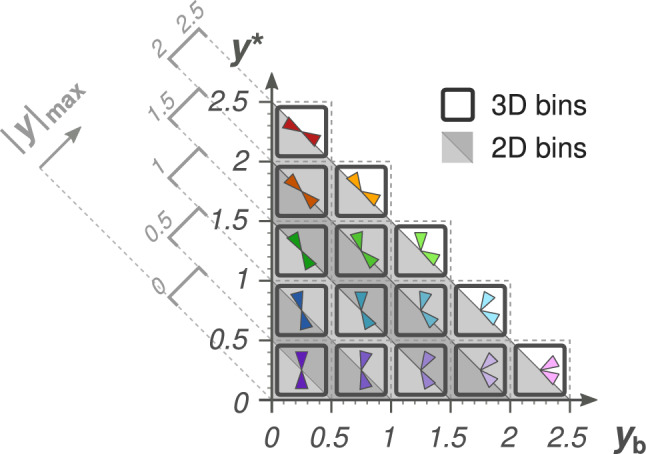
Illustration of the dijet rapidity phase space, highlighting the relationship between the variables used for the 2D and 3D measurements. The colored triangles are suggestive of the orientation of the two jets in the different phase space regions in the laboratory frame, assuming that the beam line runs horizontally

As in Eq. (4), the event yield is normalized to the observable bin widths $$\varDelta y^{*} $$, $$\varDelta y_{\text {b}} $$ and $$\varDelta x$$, where *x* stands for either $$m_{1,2} $$ or $$\langle p_{\textrm{T}} \rangle _{1,2} $$. Fifteen rapidity regions are investigated, covering the range from 0 to 2.5 in each observable, as illustrated in Fig. 1. Different invariant mass and average transverse momentum regions are measured depending on the rapidity region, covering a range of $$306< m_{1,2} < 6094\,\text {Ge}\hspace{-.08em}\text {V} $$ and $$147< \langle p_{\textrm{T}} \rangle _{1,2} < 2702\,\text {Ge}\hspace{-.08em}\text {V} $$, respectively. These ranges are obtained using an analogous procedure as for the 2D measurements.

## Unfolding

Because of the finite detector resolution and other experimental effects, such as the reconstruction efficiency, the properties of reconstructed jets differ from those of jets defined at the particle level. This leads to a migration of dijet events within the phase space spanned by the observables used for the cross section measurement. To enable a direct comparison of the measured cross sections to theoretical particle-level calculations or to other measurements, the effect of these migrations is accounted for as part of a multi-dimensional unfolding procedure.

Using simulated event samples, the dijet observables of interest are computed event by event based on both the two $p_{\textrm{T}}$-leading reconstructed jets and the jets clustered directly from generated particles. Response matrices are constructed to reflect the probability of bin-to-bin event migrations between the particle and reconstruction levels, taking all the observables used for a measurement into account simultaneously.

The measured event distributions are unfolded using the TUnfold package [29], based on the simulation-derived response matrices. While no explicit regularization of the unfolded distributions is performed, large fluctuations between neighboring bins stemming from an ill-conditioned response matrix are avoided through an appropriate choice of bins. These are chosen in such a way as to ensure that the bin sizes remain at least twice as large as the resolution in these variables, and that the purity is at the level of 50% or above. The latter is defined as the fraction of reconstructed events in each bin that originate from genuine dijet events in the same bin at the particle level.

**Fig. 2:**
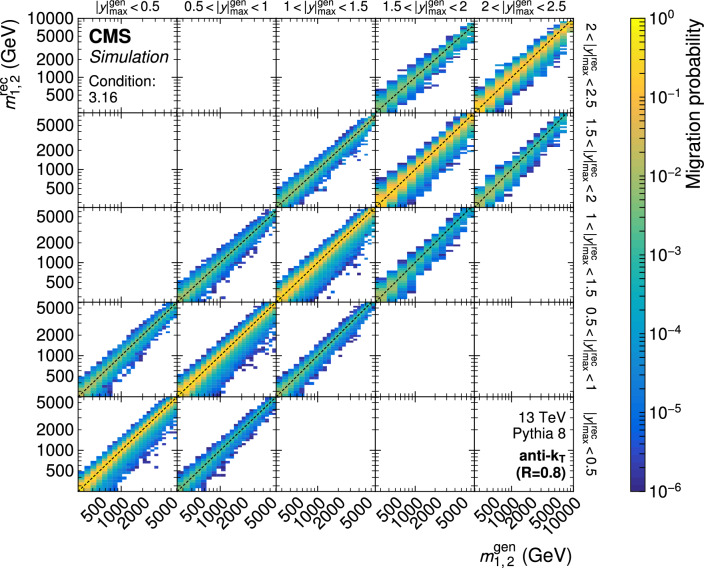
Response matrix for the 2D measurement as a function of $$m_{1,2} $$ using jets with $$R = 0.8$$. The entries represent the probability for a dijet event generated in the phase space region ($$m_{1,2} ^\text {gen}$$, $$|y |_{\text {max}} ^\text {gen}$$) indicated on the *x* axis to be reconstructed in the phase space region ($$m_{1,2} ^\text {rec}$$, $$|y |_{\text {max}} ^\text {rec}$$) indicated on the *y* axis. Response matrices for all other jet sizes and observables can be found in Appendix 1

To ensure that the unfolding problem is well-posed, a larger number of bins is chosen for the reconstructed distributions than for the particle-level distributions. Moreover, because of the larger resolution and the decrease in purity at outer rapidities, a coarser particle-level binning is chosen for the two outermost $|y |_{\text {max}}$ regions for the 2D measurements, and the corresponding nine outermost ${(y^{*} \!,~y_{\text {b}})}$ regions for the 3D measurements. All response matrices obtained in this way exhibit condition numbers of ${\approx }3$ and are thus suitable for unfolding without regularization. The condition number is defined as the absolute value of the ratio between the largest and smallest matrix eigenvalue. Figure 2 shows the responses obtained for a representative choice of jet distance parameters and dijet observables.

Aside from event migrations within the measurement phase space, contributions to each bin from spurious jet reconstructions, pileup, changes in the $p_{\textrm{T}}$ ordering of jets, or migrations into the phase space, are evaluated in the simulation and proportionally subtracted from the measured distributions prior to unfolding. Similarly, to correct for event losses due to the finite reconstruction efficiency, changes in the $p_{\textrm{T}}$ ordering of jets, or migrations outside the phase space, bin-by-bin correction factors are derived from simulation and applied to the unfolded distributions.

## Experimental uncertainties

Statistical fluctuations in the observed event counts and various systematic effects give rise to experimental uncertainties in the measured cross sections. The statistical uncertainties are calculated from the event counts in each bin assuming a Poisson distribution and the corresponding covariance matrix is propagated through the unfolding procedure to yield a full set of statistical uncertainties and correlations for the unfolded cross sections. For both the 2D and 3D measurements, the statistical uncertainties remain below 2% in most phase space regions, generally increasing to 2–5% at outer rapidities and reaching values of 20–40% at large $$m_{1,2} $$ or $$\langle p_{\textrm{T}} \rangle _{1,2} $$.

The impact of systematic effects on the cross section is generally estimated by varying experimental parameters within a $\pm 1$ standard deviation interval around the nominal value. The relative differences to the nominal result are used to construct an asymmetric confidence interval for the unfolded cross sections in each observable bin.

**Fig. 3:**
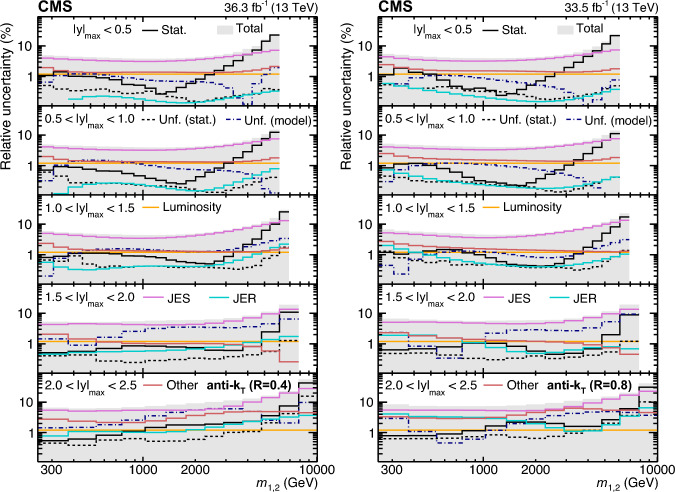
Breakdown of the experimental uncertainty for the 2D measurements as a function of $m_{1,2}$ using jets with $R = 0.4$ (left) and 0.8 (right). The individual components and abbreviations are explained in Sect. 7. The abbreviation “Unf.” refers to the unfolding uncertainties. The shaded area represents the sum in quadrature of all statistical and systematic uncertainty components

**Fig. 4:**
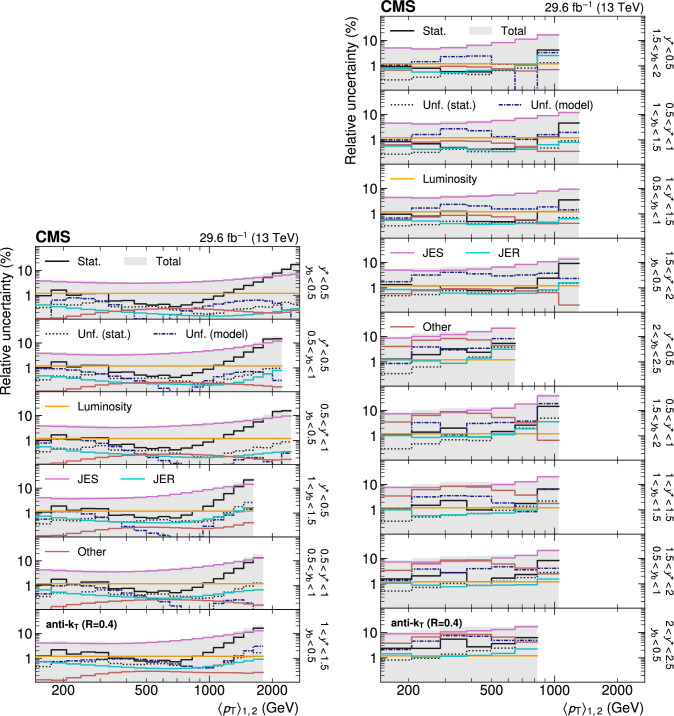
Breakdown of the experimental uncertainty for the 3D measurement as a function of $\langle p_{\textrm{T}} \rangle _{1,2}$ using jets with $R = 0.4$. The individual components and abbreviations are explained in Sect. 7. The shaded area represents the sum in quadrature of all statistical and systematic uncertainty components. Similar plots for all other jet sizes and observables can be found in Appendix B

Figures 3 and 4 show an overview of the main contributions to the experimental uncertainty in the dijet cross section for the 2D measurement for both values of *R*, and the 3D measurement as a function of $\langle p_{\textrm{T}} \rangle _{1,2}$ for $R=0.4$, respectively. The main contributions to the systematic uncertainty are due to the determination of the JES and JER, and the luminosity. A further uncertainty results from the correction of the trigger prefiring inefficiency, and is only significant in the outer rapidity regions with contributions from jets with $|y | > 2$. Other contributions, which have an overall smaller impact on the cross section, arise as a consequence of experimental methods such as unfolding. The following sections describe the individual uncertainty contributions in more detail.

### Jet energy scale uncertainty

The dominant contribution to the systematic uncertainty arises from the determination of the JES. Jets are calibrated in a multi-stage procedure to correct for experimental effects, such as contributions from pileup collisions or shifts in the jet energy due to detector or reconstruction effects. The corrections depend on the $$p_{\textrm{T}} $$ and $$\eta $$ of the jet, and lead to a total uncertainty in the energy scale of individual jets of 1–2% in the phase space considered here [30]. Since the dijet spectrum decreases exponentially as a function of $$m_{1,2}$$ and $$\langle p_{\textrm{T}} \rangle _{1,2}$$, the resulting uncertainty in the measured differential dijet cross section is amplified by this exponent. For the 2D cross sections, the JES uncertainty starts at 2–5%, reaching 30% at higher values of $$m_{1,2} $$. For the 3D cross section the total JES uncertainty increases with $$m_{1,2}$$ ($$\langle p_{\textrm{T}} \rangle _{1,2}$$) from about 3% up to values between 8 and 60% (40%), depending on the rapidity region.

The total JES uncertainty is composed of 22 individual contributions describing different systematic effects. These include, in roughly descending order of their impact on the cross section: the change in experimental conditions over time, the calibration of the relative and absolute JES as a function of $\eta$ and $p_{\textrm{T}}$, the change in response for jets initiated by gluons and different quark flavors, and pileup collisions. Each contribution represents a fully correlated uncertainty across all data points and is considered to be independent of the other contributions.

### Luminosity uncertainty

The uncertainty due to the integrated luminosity measurement is evaluated to be 1.2% [31] in all phase space regions and is considered to be fully correlated across all bins.

### Jet energy resolution uncertainty

The effect of the finite JER on the cross section is modeled using response matrices obtained from the simulation, where the effective JER is increased by factors derived from control samples in data to account for residual differences between the detector simulation and the actual data-taking conditions. The correction of the JER is applied as part of the unfolding procedure. The JER uncertainty in the cross section is estimated by performing the unfolding with response matrices derived from alternative samples, where the jet energy was smeared by factors representing a ±1 standard deviation shift in the JER compared to the nominal value. The resulting uncertainty values range from below 1% at central rapidities to at most 10% in the outer rapidity regions, and are considered to be correlated across all data points.

### Unfolding uncertainties

A further uncertainty arises as a consequence of the limited size of the simulated samples used for deriving the response matrices as part of the unfolding procedure. These are thus subject to an intrinsic statistical uncertainty, which is propagated analytically to the unfolded cross sections. In most phase space regions, this uncertainty remains below 0.5%, reaching values of 5–10% only in a small number of bins at the highest $m_{1,2}$ or $\langle p_{\textrm{T}} \rangle _{1,2}$. As an estimate of the model dependence introduced by unfolding, the difference in the cross sections unfolded with response matrices obtained from pythia  8 and MadGraph 5_amc@nlo is taken as an additional uncertainty, which is considered to be correlated across all data points. This uncertainty is typically at the level of 1%, rising up to at most 10% at outer rapidities and high $m_{1,2}$.

### Other uncertainties

The correction applied to compensate for the trigger inefficiency due to prefiring gives rise to an additional correlated uncertainty in the cross section. In general, this uncertainty is at the level of 1% or below, except in the outermost $|y |_{\text {max}}$ region and the five outermost ($y^{*}$ , $y_{\text {b}}$) regions, where it rises to about 10% (20%) at the upper end of the $m_{1,2}$ ($\langle p_{\textrm{T}} \rangle _{1,2}$) spectrum.

The uncertainty contribution from pileup interactions is determined by varying the total inelastic ${\text {p}} {\text {p}}$ cross section used for reweighting the simulated samples within its associated uncertainty of 4.6%, as obtained using the pileup counting method described in Ref. [21]. The unfolding is then performed with the resulting response matrices, taking the differences between the variations and the nominal value as a fully correlated uncertainty in the unfolded cross section, which is below 1% in all phase space regions.

The normalization uncertainty in the background contribution from spurious jet reconstructions or event migrations at the phase space boundaries is estimated to be 5% and propagated through the unfolding procedure. A further contribution to the uncertainty in the unfolded cross section is due to the correction of reconstruction inefficiencies and migrations outside the measurement phase space and is estimated to be 5% of the corresponding correction factors. Each of the above contributions is considered to be fully correlated across all data points.

## Theoretical predictions

Fixed-order theoretical predictions for the 2D and 3D dijet cross sections are obtained up to NNLO accuracy in pQCD with the nnlojet program (revision 5918) [32]. The nnlojet program is interfaced to fastNLO (version 2.3) [33, 34] via the APPLfast interface (version 0.0.46) [35, 36] to provide interpolation grids that allow theoretical predictions to be obtained for arbitrary PDFs and for different values of the renormalization scale $\mu _{\textrm{R}}$, the factorization scale $\mu _{\textrm{F}}$, and the strong coupling constant $\alpha _\textrm{S} (m_{{\text {Z}}})$, without the need to repeat the full calculation.

**Fig. 5:**
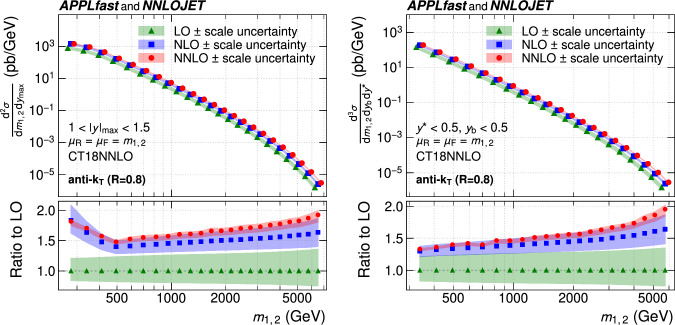
Theoretical predictions for the 2D (upper) and 3D (lower) cross sections, as a function of $m_{1,2}$, illustrated here in the rapidity regions $1.0<|y |_{\text {max}} <1.5$ and $y_{\text {b}} < 0.5$, $y^{*} < 0.5$, together with the corresponding six-point scale uncertainty for $\mu _{\textrm{R}} =\mu _{\textrm{F}} =m_{1,2}$ using the CT18 NNLO PDF set. In the upper panels, the curves and symbols are slightly shifted for better visibility. The lower panels show the ratio to the respective prediction at LO. The fluctuations in the NNLO predictions are due to the limited statistical precision of the calculation

Following recommendations outlined in Ref. [3], $$m_{1,2} $$ is chosen as the central reference value for both $$\mu _{\textrm{R}} $$ and $$\mu _{\textrm{F}} $$. To estimate the theoretical uncertainty due to missing higher-order terms in perturbation theory, the conventional recipe [37–39] of varying the $$\mu _{\textrm{R}}$$ and $$\mu _{\textrm{F}}$$ scales is applied. More precisely, the so-called scale uncertainty is derived from the envelope of the theoretical predictions obtained for the six scale variations corresponding to ($$\mu _{\textrm{R}}/m_{1,2} $$, $$\mu _{\textrm{F}}/m_{1,2} $$) = (1/2, 1/2), (1/2, 1), (1, 1/2), (2, 1), (1, 2), and (2, 2). As an example, Fig. 5 shows the resulting uncertainty for theoretical predictions of the 2D and 3D dijet cross sections obtained at LO, next-to-leading order (NLO), and NNLO. In most phase space regions, the NLO and NNLO scale uncertainty bands overlap, indicating good perturbative convergence. However, towards small values of $$m_{1,2} $$ and large rapidity separations $$y^{*}$$, a steep rise in the ratio of the higher-order predictions with respect to LO, referred to here as the *K* factors, is observed, leading to a reduced overlap and an increased scale uncertainty. For an ideal dijet event with two jets of equal $$p_{\textrm{T}}$$ produced in a back-to-back configuration, the dijet invariant mass is given by $$m_{1,2} = 2p_{\textrm{T}} \cosh (y^{*})$$. The rise in the *K* factors then is understood to be caused by the minimum $$p_{\textrm{T}}$$ requirements imposed on the two leading jets, which at small dijet mass restrict the phase space accessible to LO processes in favor of higher-order contributions.

The NNLO contribution is based on the leading-color and leading-flavor-number approximation [3, 4]. Subleading-color contributions have been shown to be at the percent level at NLO and are expected to be even smaller in comparison to the leading-color result at NNLO [40]. It is worth noting, however, that in a recent investigation [41] a significant impact of subleading-color contributions was found for the NNLO prediction of the $\langle p_{\textrm{T}} \rangle _{1,2}$-dependent 3D CMS dijet measurement at $\sqrt{s} = 8\,\text {Te}\hspace{-.08em}\text {V}$ with a jet distance parameter $R = 0.7$ [5]. The reported effect can lead to a decrease in the cross section of up to 5 for small $\langle p_{\textrm{T}} \rangle _{1,2}$ and an increase of up to 3% for large $\langle p_{\textrm{T}} \rangle _{1,2}$, which is beyond the size of the scale uncertainty at NNLO. For the CMS inclusive jet measurement at $\sqrt{s} = 13\,\text {Te}\hspace{-.08em}\text {V}$ [42] with jet distance parameters of $R = 0.4$ and 0.7, the effect was determined to be much smaller and to be covered by scale uncertainty estimates, except towards small jet $p_{\textrm{T}}$ for the smaller jet size. Predictions for the 2D dijet measurement performed as a function of $m_{1,2}$ and $y^{*}$ at $\sqrt{s} = 7\,\text {Te}\hspace{-.08em}\text {V}$ by the ATLAS Collaboration [11] were also less affected even with $R = 0.4$. The effect on the dijet observables under examination here is not yet known. The 2D and 3D measurements presented here for two jet distance parameters, $R=0.4$ and 0.8, and for the two dijet observables $m_{1,2}$ and $\langle p_{\textrm{T}} \rangle _{1,2}$, provide an ideal set of measurements to further study the impact of subleading-color corrections in comparison to data.

To compare with CMS data unfolded to the particle level, the fixed-order predictions are complemented by nonperturbative (NP) correction factors $c_{\textrm{NP}}$, which are defined as the ratio between the nominal cross sections with and without multiple parton interactions (MPI) and hadronization (HAD) effects, as given by a chosen MC event generator,

6 $$\begin{aligned} c_{\textrm{NP}} = \frac{\sigma ^\text {PS+MPI+HAD}}{\sigma ^\text {PS}}, \end{aligned}$$

where the parton shower (PS) is considered to be a perturbative component.

The model dependence of the NP corrections is evaluated by comparing results from several MC event generators. Leading-order particle-level predictions are obtained from pythia (version 8.240), using the tunes CUETP8M1 [18] and CUETP8M2T4 [43], and herwig++  [44] (version 2.7.1) using the EE5C tune [45]. These generators are interfaced to powheg  [46–49] (version 2J V2_Mar2016) to provide NLO predictions. An additional set of predictions is obtained from herwig  7 [50] (version 7.2.2) with the CH3 tune [51] at both LO and NLO.

To mitigate statistical fluctuations, the corrections are parametrized by a smooth function $$f(x) = a / x^b + c$$, where *x* is either $$m_{1,2} $$ or $$\langle p_{\textrm{T}} \rangle _{1,2} $$. The parameters *a*, *b*, and *c* are obtained in a least-squares fit to the binwise correction factors $$c_{\textrm{NP}} $$ obtained from Eq. (6) in each rapidity region. The fits provide a good description of the correction factors in most phase space regions. For a number of low-$$m_{1,2}$$ bins, where the phase space is constrained by the minimum $$p_{\textrm{T}}$$ requirements on the two leading jets, the value of $$c_{\textrm{NP}} $$ is taken directly as the correction factor. The final correction factor in each bin is obtained as the midpoint between the largest and smallest value of $$c_{\textrm{NP}} $$ obtained across all MC configurations, and half the difference between the largest and smallest value is assigned as an uncertainty.

**Fig. 6:**
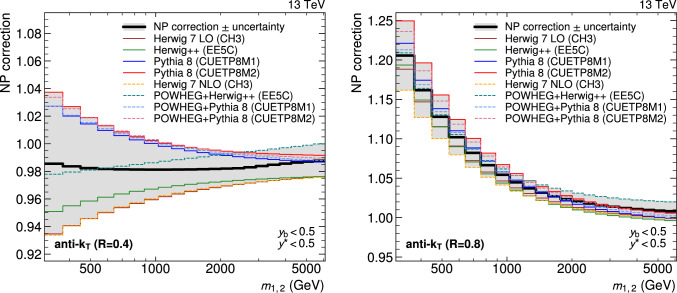
Nonperturbative correction factors obtained for jets with $$R = 0.4$$ (upper) and 0.8 (lower) as a function of $$m_{1,2}$$, illustrated here in the rapidity region ($$y_{\text {b}} < 0.5$$, $$y^{*} < 0.5$$). Individual correction factors are first derived from simulation using eight different MC configurations. The largest and smallest value obtained in each observable bin is then used to define the final correction factor and its associated uncertainty. The correction values are larger for jets with $$R = 0.8$$, increasing to over 20% in the lowest $$m_{1,2} $$ bin

The resulting NP corrections are illustrated in Fig. 6. For jets with $R = 0.4$, the contributions from hadronization and MPI largely cancel, leading to NP corrections compatible with unity within their uncertainty. In contrast, the MPI contribution dominates for jets with $R = 0.8$, resulting in significantly larger NP corrections of $\approx$20% at low values of $m_{1,2}$. The size of the uncertainty is similar for both jet sizes.

It is also observed that the NP corrections obtained with pythia  8 are in general larger than those from herwig++ or herwig  7, such that this difference is the dominant contribution to the NP correction uncertainty. While some dependence on the tune is observed when comparing the predictions from CUETP8M1 and CUETP8M2T4, the impact is typically small. In most cases, the values obtained at NLO are seen to be comparable to those obtained at LO from the same generator, with the notable exception of herwig++ , where the NLO result obtained using powheg is consistently higher than the LO result.

**Fig. 7:**
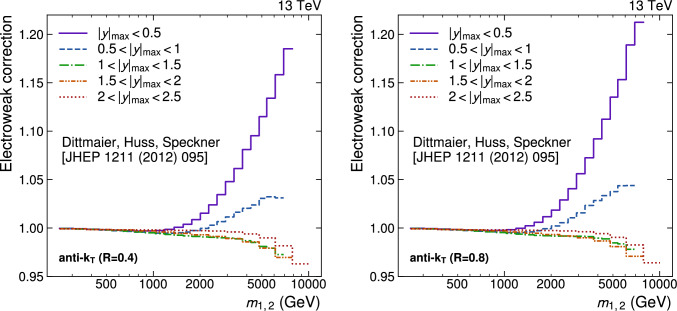
Electroweak correction factors obtained for jets with $R = 0.4$ (upper) and 0.8 (lower) as a function of $m_{1,2}$ in the five different $|y |_{\text {max}}$ regions. The corrections depend strongly on the kinematic properties of the jets and are observed to be largest at central rapidities for $m_{1,2} > 1\,\text {Te}\hspace{-.08em}\text {V}$

For jet transverse momenta in the $\text {Te}\hspace{-.08em}\text {V}$ range, electroweak contributions to the differential dijet cross section become important and must be considered in addition to the NNLO pQCD calculation [52]. These effects, which arise from the virtual exchange of soft or collinear W or Z bosons, are accounted for by applying a multiplicative correction factor to the pQCD prediction. As shown in Fig. 7, these factors exhibit a strong dependence on the rapidity and $p_{\textrm{T}}$ of the jets. Particularly at small $|y |_{\text {max}}$ or $y^{*}$, the electroweak correction reaches 10–20% for dijet masses beyond 5$\,\text {Te}\hspace{-.08em}\text {V}$, where experimental uncertainties become large as well. The uncertainty on this correction is therefore considered to be negligible with respect to other large uncertainties.

**Fig. 8:**
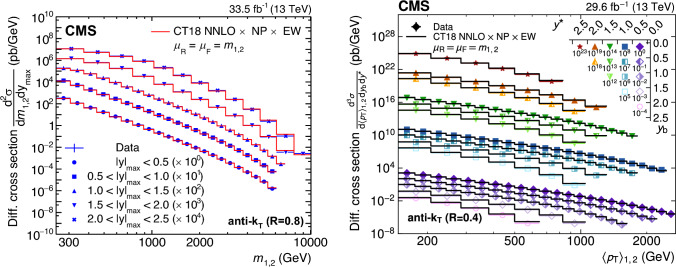
Differential dijet cross sections, illustrated here for the 2D measurement as a function of $$m_{1,2} $$ using jets with $$R = 0.8$$ (upper), and the 3D measurement as a function of $$\langle p_{\textrm{T}} \rangle _{1,2} $$ using jets with $$R = 0.4$$ (lower). The markers and lines indicate the measured unfolded cross sections and the corresponding NNLO predictions, respectively. For better visibility, the values are scaled by a factor depending on the rapidity region, as indicated in the legend. Analogous plots for all other jet sizes and observables can be found in Appendix  B

**Fig. 9:**
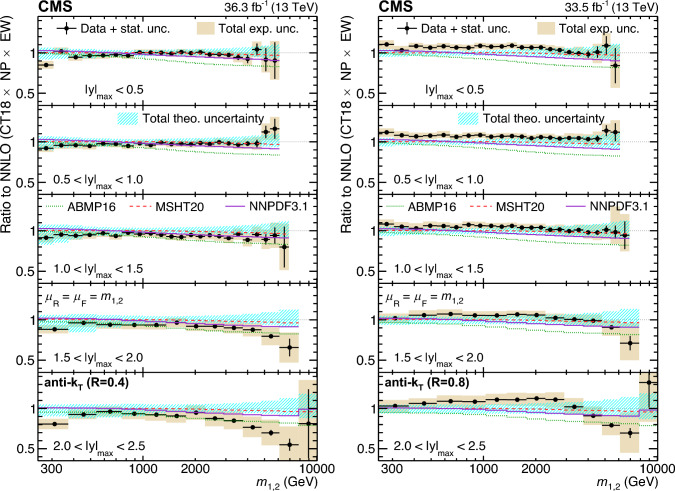
Comparison of the 2D dijet cross section as a function of $$m_{1,2} $$ to fixed-order theoretical calculations at NNLO, using jets with $$R = 0.4$$ (left) and 0.8 (right). Shown are the ratios of the measured cross sections (markers) to the predictions obtained using the CT18 NNLO PDF set. The error bars and shaded yellow regions indicate the statistical and the total experimental uncertainties of the data, respectively, and the hatched teal band indicates the sum in quadrature of the PDF, NP, and scale uncertainties. Alternative theoretical predictions obtained using other global PDF sets are shown as colored lines. Similar plots for the individual rapidity regions can be found in Appendix B

**Fig. 10:**
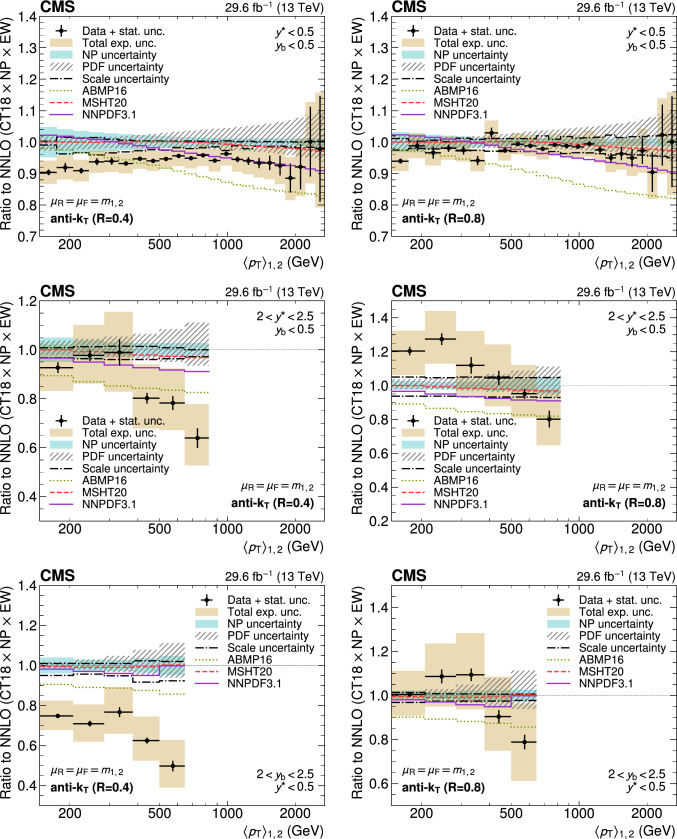
Comparison of the 3D dijet cross section for jets with $R = 0.4$ (left) and 0.8 (right) as a function of $\langle p_{\textrm{T}} \rangle _{1,2}$ to fixed-order theoretical calculations at NNLO, shown here for three out of the total of 15 rapidity regions. The data points and predictions for alternative PDFs are analogous to those in Fig. 9. In addition, the separate contributions to the theory uncertainty due to the CT18 PDFs, NP corrections, and six-point scale variations are shown explicitly. Similar plots for all rapidity regions and observables can be found in Appendix  B

## Comparison to theory

An overview of the unfolded cross sections obtained for the 2D and 3D measurements and the corresponding fixed-order theoretical predictions at NNLO, complemented by NP and electroweak corrections, is presented in Fig. 8. For a more detailed comparison, ratios of the measured cross sections to the theoretical predictions are shown in Figs. 9 and 10.

The theoretical predictions are obtained using recent NNLO PDF sets available via the lhapdf  [53] library (version 6.3.0), namely ABMP16 [54], CT18 [55], MSHT20 [56], and NNPDF3.1 [57]. All PDF sets are derived in global fits to data from multiple experiments while fixing the value of the strong coupling constant $\alpha _\textrm{S} (m_{{\text {Z}}})$ to 0.118, except for ABMP16, where $\alpha _\textrm{S} (m_{{\text {Z}}}) = 0.1147$ is determined in the fit together with all other parameters. The uncertainties in the cross section predictions due to the PDFs are calculated as 68% confidence intervals following the prescriptions given in the respective references. The PDF uncertainty bands shown in Fig. 10 are obtained using the CT18 PDF set and do not account for the finite precision of $\alpha _\textrm{S} (m_{{\text {Z}}})$.

The predictions for different PDFs are generally in agreement with each other within the PDF uncertainties, except for the AMBP16 PDF, for which the predicted cross sections are generally smaller than those for other PDFs. At large $$m_{1,2} $$ or $$\langle p_{\textrm{T}} \rangle _{1,2} $$, the predictions obtained for the different PDF sets show a diverging trend, while still remaining compatible within the PDF uncertainties.

The level of agreement between the theoretical predictions and the data is observed to be good in most phase space regions, with some deviations at the lower ends of the spectra and in the outer rapidity regions. In general, the theoretical predictions for $R = 0.8$ are observed to provide a better description of the data than for $R = 0.4$, which is consistent with past observations [42, 58–61].

## The QCD analysis

To evaluate the impact of the present measurements on determinations of the proton PDFs and the strong coupling constant, a QCD analysis is performed following the approach taken by earlier HERAPDF analyses [1, 2, 62]. The data used in the QCD analysis comprise DIS measurements [1, 2] obtained in ${\text {e}} ^\pm {\text {p}}$ collisions at the HERA collider experiments H1 and ZEUS as a function of the momentum transfer $Q^2$, supplemented by the present measurements of the dijet cross section.

The HERA measurements correspond to charged-current (CC) DIS data collected in ${\text {e}} ^{-} {\text {p}}$ and ${\text {e}} ^{+} {\text {p}}$ collisions at a proton beam energy of $E_{{\text {p}}} = 920\,\text {Ge}\hspace{-.08em}\text {V}$, and neutral-current (NC) DIS data collected in ${\text {e}} ^{+} {\text {p}}$ collisions at proton beam energies of $E_{{\text {p}}} = 460$, 575, 820, and 920$\,\text {Ge}\hspace{-.08em}\text {V}$. Only data points with $Q^2$ values above a threshold $Q^2_\text {min} = 10\,\text {Ge}\hspace{-.08em}\text {V} ^2$ are included, to ensure a good description of the measurements by the theoretical predictions.

It is well known that fixed-order predictions work most reliably for inclusive observables, i.e., where the phase space for QCD radiation is not restricted. Such restrictions – as for example the choice of a small distance parameter *R* for jet clustering – introduce disparities in the cancellation of singularities between real emissions and virtual corrections, leading to large logarithmic terms that have a negative impact on the perturbative convergence and would require resummation [63]. Moreover, as discussed in Sect. 8, there are indications that subleading-color corrections might have a smaller impact for larger values of *R*, and for $m_{1,2}$ as compared to $\langle p_{\textrm{T}} \rangle _{1,2}$. Thus, to profit most from the available predictions at NNLO, only the dijet cross sections as a function of $m_{1,2}$ for the larger value of $R = 0.8$ are used in the QCD analysis.

Theoretical predictions for the dijet cross sections are obtained from nnlojet and fastNLO as interpolation grids at NNLO accuracy, taking into account the full dependence of the NNLO cross sections on the PDFs, the strong coupling constant, $$\mu _{\textrm{R}} $$, and $$\mu _{\textrm{F}} $$. Following Ref. [3], the dijet invariant mass $$m_{1,2} $$ is chosen as the central value for both $$\mu _{\textrm{R}} $$ and $$\mu _{\textrm{F}} $$. The cross sections are corrected additionally for NP and electroweak effects as described in Sect. 8.

Simultaneous determinations of PDFs and the strong coupling constant at the scale of the Z  boson mass, $\alpha _\textrm{S} (m_{{\text {Z}}})$, are performed with the xFitter program (version 2.0.1) [64, 65], using the HERA data together with either the 2D or 3D dijet cross sections as inputs. Access to the theoretical predictions for the dijet cross sections is provided by fastNLO. The evolution of PDFs following the DGLAP equations [66–68] is performed using the qcdnum package (version 17-01/15) [69]. Contributions from heavy quarks are treated in the Thorne–Roberts optimal variable flavor number scheme (RTOPT) [70–72], with the masses of the charm and bottom quarks set to $m_{\text {c}} = 1.43\,\text {Ge}\hspace{-.08em}\text {V}$ and $m_{\text {b}} = 4.5\,\text {Ge}\hspace{-.08em}\text {V}$, respectively.

In the HERAPDF approach, the proton structure is expressed in terms of the gluon distribution $\text {g}(x)$, the up and down valence quark distributions $\text {u}_\text {v}(x)$ and $\text {d}_\text {v}(x)$, and the up- and down-type sea antiquark distributions $\overline{\textrm{U}} (x)$ and $\overline{\textrm{D}} (x)$. For each of these distributions $f\!$, the dependence on the proton momentum fraction *x* carried by a parton is parametrized at a starting scale $\mu _{\textrm{F},\,0} ^2 = 1.9\,\text {Ge}\hspace{-.08em}\text {V} ^2$ as

7 $$\begin{aligned} x\,{\!f}(x, \mu _{\textrm{F},\,0} ^2) = A_{\!f}\,x^{B_{\!f}}\,(1 - x)^{C_{\!f}}\,(1 + D_{\!f}\,x + E_{\!f}\,x^2). \end{aligned}$$

The overall normalization of the PDFs is given by the *A* parameters, with $A_{\text {u}_\text {v}}$, $A_{\text {d}_\text {v}}$, and $A_{\text {g}}$ being constrained by the quark number and momentum sum rules. The *B* and *C* parameters control the shape of the distribution as *x* approaches the edges of its domain at 0 and 1, respectively. The *D* and *E* parameters represent additional degrees of freedom related to the functional forms.

The sea quark contributions are given by $\overline{\textrm{U}} (x) = \overline{\textrm{u}} (x)$ and $\overline{\textrm{D}} (x) = \overline{\textrm{d}} (x) + \overline{\textrm{s}} (x)$, where $\overline{\textrm{u}} (x)$, $\overline{\textrm{d}} (x)$, and $\overline{\textrm{s}} (x)$ refer to the distribution of up, down, and strange antiquarks, respectively. A fixed overall normalization ratio is imposed by the requirement $A_{\overline{\textrm{U}}} = A_{\overline{\textrm{D}}} (1 - f_\textrm{s})$, where the strangeness fraction is given by $f_\textrm{s} = \overline{\textrm{s}}/(\overline{\textrm{s}} + \overline{\textrm{d}})$ and set to 0.4 following Ref. [2]. To enforce a similar behavior of the quark sea as $x \rightarrow 0$, the requirement $B_{\overline{\textrm{U}}} = B_{\overline{\textrm{D}}}$ is imposed. The total sea quark distribution is defined as $\varSigma (x) = 2\left( \overline{\textrm{U}} (x) + \overline{\textrm{D}} (x)\right)$.

The above constraints result in a total of ten *A*, *B*, and *C* parameters whose values are determined during the fit procedure. Additional *D* and *E* parameters are included where it is found that these lead to an improved fit quality in terms of the total $\chi ^2$ value, following the procedure outlined in Ref. [2]. Starting from the initial parametrization with no additional parameters, the change in $\chi ^2$ resulting from the inclusion of any of the remaining *D* and *E* parameters in the fit is evaluated. The parameter resulting in the largest decrease in $\chi ^2$ is added to the parametrization, and the procedure is repeated until no further significant improvement is observed. The final parametrization obtained in this way for the fits including the CMS dijet measurements is given by:

8 $$\begin{aligned} \begin{aligned} x\,{\text {g}}(x, \mu _{\textrm{F},\,0} ^2)&= A_{\text {g}}\,x^{B_{\text {g}}}(1 - x)^{C_{\text {g}}},\\ x\,{\text {u}_\text {v}}(x, \mu _{\textrm{F},\,0} ^2)&= A_{\text {u}_\text {v}}\,x^{B_{\text {u}_\text {v}}}(1 - x)^{C_{\text {u}_\text {v}}}(1 + D_{\text {u}_\text {v}}x + E_{\text {u}_\text {v}}x^2),\\ x\,{\text {d}_\text {v}}(x, \mu _{\textrm{F},\,0} ^2)&= A_{\text {d}_\text {v}}\,x^{B_{\text {d}_\text {v}}}(1 - x)^{C_{\text {d}_\text {v}}},\\ x\,{\overline{\textrm{U}}}(x, \mu _{\textrm{F},\,0} ^2)&= A_{\overline{\textrm{U}}}\,x^{B_{\overline{\textrm{U}}}}(1 - x)^{C_{\overline{\textrm{U}}}}\,(1 + D_{\overline{\textrm{U}}}\,x),\\ x\,{\overline{\textrm{D}}}(x, \mu _{\textrm{F},\,0} ^2)&= A_{\overline{\textrm{D}}}\,x^{B_{\overline{\textrm{D}}}}(1 - x)^{C_{\overline{\textrm{D}}}}. \end{aligned} \end{aligned}$$

Uncertainties in the fitted PDFs are determined using a similar procedure as the one described in Ref. [62]. Separate contributions for *fit*, *model*, *scale*, and *parametrization* uncertainties are obtained as described in the following.

The fit uncertainty represents the propagation to the PDFs of the uncertainties in the input measurements, theoretical predictions, and theory correction factors. It is estimated following the MC method outlined in Refs. [73, 74], whereby a large number of alternative fits (MC replicas) are performed with random variations of the input data according to their statistical and systematic uncertainties, taking the standard deviation of the resulting PDFs as an estimate of the fit uncertainty.

An alternative estimate for the fit uncertainty is obtained via the Hessian method [75] and found to be comparable to the MC fit uncertainty in most cases, apart from the $\text {u}_\text {v}$ distribution, where it is found that the fit uncertainty is significantly underestimated by the Hessian method at $x < 0.1$. This is understood to be a consequence of the more flexible parametrization of $\text {u}_\text {v}$ resulting from the parametrization scan, which is driven by the high-*x* region where the input data are constraining.

The model uncertainty arises from the choices made for the values of certain non-PDF parameters: the minimum $Q^2$ value used for restricting the HERA data, the strangeness fraction $f_\textrm{s}$, the charm and bottom quark masses $m_\text {c}$ and $m_\text {b}$, and the value of the starting scale $\mu _{\textrm{F},\,0}$. It is estimated by varying the values of these parameters up and down from their nominal values as indicated in Table 2, and adding the differences to the nominal fit result in quadrature separately for each variation direction.

**Table 2:** Nominal values and variations of parameters used to determine the PDF model uncertainty. Variations marked with an asterisk are in conflict with the requirement $\mu _{\textrm{F},\,0} < m_{\text {c}}$ and thus cannot be used directly for the uncertainty estimation. Following Ref. [62], the results obtained for the opposite variation are symmetrized in these cases

Parameter	Nominal value	Variations	
		*down*	*up*
$Q^2_\text {min}$ ($\text {Ge}\hspace{-.08em}\text {V} ^2$)	10	7.5	12.5
$f_\textrm{s}$	0.4	0.3	0.5
$m_{\text {c}}$ ($\text {Ge}\hspace{-.08em}\text {V}$ )	1.43	1.37*	1.49
$m_{\text {b}}$ ($\text {Ge}\hspace{-.08em}\text {V}$ )	4.5	4.25	4.75
$\mu _{\textrm{F},\,0} ^2$ ($\text {Ge}\hspace{-.08em}\text {V} ^2$)	1.9	1.6	2.2*

A further uncertainty arises because of the choice of PDF parametrization. It is estimated by performing alternative fits that include one additional *D* or *E* parameter compared to the nominal parametrization. The maximum deviation between the nominal PDF and those obtained from the alternative parametrizations is taken as an additional parametrization uncertainty.

**Table 3:** Goodness-of-fit values for the fits to the HERA DIS data alone, and together with the CMS dijet measurements, using the PDF parametrization given in Eq. 8. The table shows the partial $\chi ^2$ values divided by the number of data points for the HERA DIS datasets and each of the dijet rapidity regions. The total $\chi ^2$ value, divided by the number of degrees of freedom, is given at the bottom of the table

		Partial $\chi ^2$ / $n_\text {data}$		
		HERA DIS	HERA DIS + CMS 13$\,\text {Te}\hspace{-.08em}\text {V}$ dijets	
Data set			2D	3D
CMS dijet 2D				
$|y |_{\text {max}} < 0.5$			18 / 22	
$0.5< |y |_{\text {max}} < 1$			15 / 22	
$1< |y |_{\text {max}} < 1.5$			16 / 23	
$1.5< |y |_{\text {max}} < 2$			15 / 12	
CMS dijet 3D				
$y_{\text {b}} < 0.5$,	$y^{*} < 0.5$			22 / 21
$y_{\text {b}} < 0.5$,	$0.5< y^{*} < 1$			24 / 19
$0.5< y_{\text {b}} < 1$,	$y^{*} < 0.5$			49 / 19
$0.5< y_{\text {b}} < 1$,	$0.5< y^{*} < 1$			13 / 17
$0.5< y_{\text {b}} < 1$,	$1< y^{*} < 1.5$			8 / 7
$1< y_{\text {b}} < 1.5$,	$0.5< y^{*} < 1$			10 / 7
$0.5< y_{\text {b}} < 1$,	$1.5< y^{*} < 2$			9 / 6
$1< y_{\text {b}} < 1.5$,	$1< y^{*} < 1.5$			4 / 6
$1.5< y_{\text {b}} < 2$,	$0.5< y^{*} < 1$			8 / 5
HERA1+2				
CC ${\text {e}} ^{-} {\text {p}}$, $E_\text {p} = 920\,\text {Ge}\hspace{-.08em}\text {V}$		51 / 42	51 / 42	50 / 42
CC ${\text {e}} ^{+} {\text {p}}$, $E_\text {p} = 920\,\text {Ge}\hspace{-.08em}\text {V}$		37 / 39	37 / 39	38 / 39
NC ${\text {e}} ^{-} {\text {p}}$, $E_\text {p} = 920\,\text {Ge}\hspace{-.08em}\text {V}$		221 / 159	222 / 159	221 / 159
NC ${\text {e}} ^{+} {\text {p}}$, $E_\text {p} = 460\,\text {Ge}\hspace{-.08em}\text {V}$		198 / 177	197 / 177	198 / 177
NC ${\text {e}} ^{+} {\text {p}}$, $E_\text {p} = 575\,\text {Ge}\hspace{-.08em}\text {V}$		186 / 221	186 / 221	186 / 221
NC ${\text {e}} ^{+} {\text {p}}$, $E_\text {p} = 820\,\text {Ge}\hspace{-.08em}\text {V}$		55 / 61	55 / 61	55 / 61
NC ${\text {e}} ^{+} {\text {p}}$, $E_\text {p} = 920\,\text {Ge}\hspace{-.08em}\text {V}$		359 / 317	364 / 317	362 / 317
Total $\chi ^2/n_\text {dof}$		1161 / 1003	1232 / 1081	1339 / 1109

Finally, a scale uncertainty is estimated to account for missing higher orders in perturbation theory by varying $\mu _{\textrm{R}}$ and $\mu _{\textrm{F}}$ as described in Sect. 8. The envelope of the PDFs obtained with these alternative scale choices is defined as the scale uncertainty.

As discussed in Sect. 9, the level of agreement between the data and the theoretical predictions obtained with various global PDF sets varies according to the phase space region and is generally worse at outer rapidities. For the PDF determinations performed using the present data, a poor fit quality is observed in a small number of rapidity regions at high $$|y |_{\text {max}} $$, $$y^{*} $$ or $$y_{\text {b}} $$, with the partial $$\chi ^2$$ divided by the number of data points reaching values of $$\approx $$3.

The effect of including these regions in the PDF determinations is investigated by comparing to fits performed with only a subset of rapidity regions, in which the data are well described by the theoretical predictions. While this results in an increased fit uncertainty, a sizable reduction in the parametrization uncertainty – and to a lesser extent the scale uncertainty – is achieved for the restricted fits. Consequently, the fit results are derived with the chosen subset of rapidity regions, which are indicated in Table 3 along with the total and partial $\chi ^2$ values, which are close to unity in most rapidity regions, except for the bin $0.5< y_{\text {b}} < 1$, $y^{*} < 0.5$. The results of fits including all rapidity regions are provided for reference in Appendix A.

**Fig. 11:**
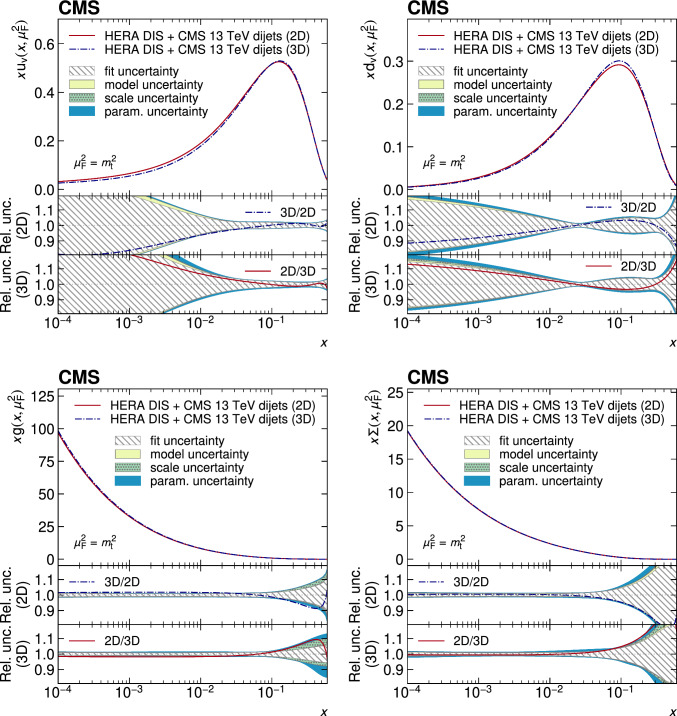
Parton distributions obtained in a fit to HERA DIS data together with the CMS 2D or 3D dijet measurements. The top panels show the PDFs of the up and down valence quarks (upper row), of the gluon (lower left), and of the total sea quarks (lower right) as a function of the fractional parton momentum *x* at a factorization scale equal to the top quark mass. The middle (lower) panels show the relative uncertainty contributions obtained for the 2D (3D) fit, as well as the ratios of the fitted central values

**Fig. 12:**
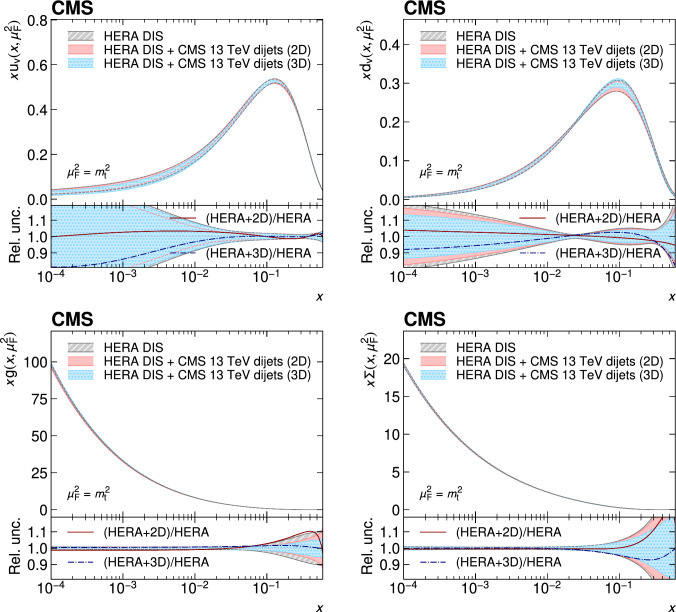
Parton distributions obtained in a fit to HERA DIS data together with the CMS dijet data, compared to a fit to HERA DIS data alone. Shown are the PDFs of the up and down valence quarks (upper row), of the gluon (lower left), and of the total sea quarks (lower right) as a function of the fractional parton momentum *x* at a factorization scale equal to the top quark mass. The bands indicate the fit uncertainty and are shown in the lower panels as a relative uncertainty with respect to the corresponding central values. The lines in the lower panels show the ratios between the fitted central values

The PDFs resulting from the fits including the CMS dijet measurements are shown in Fig. 11, along with the different uncertainty contributions. The PDFs obtained with the inclusion of the 2D data are compatible with those obtained from the 3D data within the total uncertainty, which is obtained by adding together the parametrization uncertainty and the sum in quadrature of the fit, model, and scale uncertainty contributions. For most of the distributions, a smaller fit uncertainty is obtained in the 3D fit compared to the 2D one, while the model uncertainty is of a similar size, and the scale and parametrization uncertainties are slightly larger for the 3D fit in certain *x* regions.

To evaluate the impact of the present measurements on the PDF determination, fits are performed using the HERA DIS data alone, using the same PDF parametrization as for the fits including the dijet measurements. Figure 12 shows a comparison between the PDFs obtained using only the HERA DIS data, and those obtained when fitting the CMS dijet data in addition, along with the respective fit uncertainties. The distributions obtained with and without the inclusion of the dijet measurements are observed to be compatible with each other, and a general reduction in the fit uncertainty is observed when the CMS data is included in the fit. In particular, the precision of the gluon PDF is improved for parton momentum fractions $x > 0.1$, where the uncertainty is reduced by up to a factor of $\approx$2 by the inclusion of the dijet measurements. The 3D data are observed to constrain the gluon PDF to higher values of *x* compared to the 2D data.

For the PDF determinations presented above, the value of $\alpha _\textrm{S} (m_{{\text {Z}}})$ is extracted from the data by including it in the fits as a free parameter, thus ensuring a consistent treatment of correlations between $\alpha _\textrm{S} (m_{{\text {Z}}})$ and the PDF parameters, in particular those of the gluon distribution. The value of $\alpha _\textrm{S} (m_{{\text {Z}}})$ obtained in the fit to the 2D dijet cross sections is 
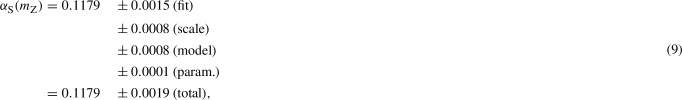


where the central value (fit uncertainty) is obtained as the average (standard deviation) over the ensemble of MC replicas. The remaining uncertainties are determined analogously to the PDFs, and in particular the parametrization uncertainty contributes linearly to the total uncertainty while the remaining contributions are added in quadrature. For the 3D dijet measurement, the result obtained is 
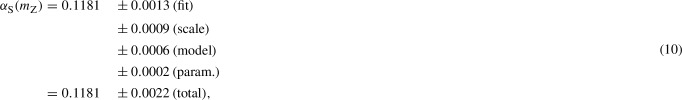
 which is in good agreement with the 2D result.

The values of $\alpha _\textrm{S} (m_{{\text {Z}}})$ determined from the dijet measurements are in agreement with the value of $0.1166\pm 0.0017$ obtained in Ref. [61], and with the world average value of $0.1179\pm 0.0009$ [76].

Parton distributions obtained in previous analyses at $\sqrt{s}$ = 8 or 13$\,\text {Te}\hspace{-.08em}\text {V}$ of the inclusive jet [60, 61, 77] or the 3D dijet cross section [5] are not easily comparable directly because of significant differences in the fit setup, the PDF parametrizations, the model parameters, and particularly in the theoretical calculations at 8$\,\text {Te}\hspace{-.08em}\text {V}$, which were only available at NLO. Taking the fit uncertainty in $\alpha _\textrm{S} (m_{{\text {Z}}})$ obtained in a simultaneous fit with the PDFs as a figure of merit, the 13$\,\text {Te}\hspace{-.08em}\text {V}$ results are more precise, which is consistent with the increase in integrated luminosity.

## Summary

The dijet production cross section is measured based on ${\text {p}} {\text {p}}$ collision data recorded by the CMS detector in 2016 at $\sqrt{s} = 13\,\text {Te}\hspace{-.08em}\text {V}$, corresponding to an integrated luminosity of up to 36.3$\,\text {fb}^{-1}$.

The measurements are performed double-differentially (2D) as a function of the dijet invariant mass $m_{1,2}$ in five regions of the maximal absolute rapidity $|y |_{\text {max}}$ of the two jets with the largest transverse momenta, and triple-differentially (3D) as a function of either $m_{1,2}$ or the average transverse momentum $\langle p_{\textrm{T}} \rangle _{1,2}$ in 15 bins of the rapidity variables $y^{*}$ and $y_{\text {b}}$. The latter two variables correspond to the rapidity separation of the two jets, and the total boost of the dijet system, respectively. All measurements are performed for jets clustered using the anti-$k_{\textrm{T}}$ jet algorithm with distance parameters $R=0.4$ and 0.8, and the cross sections are unfolded in all measurement dimensions simultaneously to correct for detector effects.

This is the first time that such a large set of multidifferential dijet measurements for two observables, $\langle p_{\textrm{T}} \rangle _{1,2}$ and $m_{1,2}$, and two jet distance parameters, $R = 0.4$ and 0.8, is made available for comparison to theory and use in fits of the parton distribution functions (PDFs) of the proton. Predictions at next-to-next-to-leading order (NNLO) in perturbative quantum chromodynamics, supplemented with electroweak and nonperturbative corrections are observed to describe the data better for $R = 0.8$.

Using the measurement of $m_{1,2}$ for $R = 0.8$, the PDFs of the proton are determined simultaneously in fits to the dijet measurements together with deep-inelastic scattering data from the HERA experiments following the approach described in earlier HERAPDF analyses [1, 2, 62]. The results obtained from the double- and triple-differential measurements are compatible within the estimated uncertainties. The inclusion of either of the dijet measurements leads to an improved determination of the PDFs compared to fits to HERA data alone. In particular, the uncertainty in the gluon distribution at fractional proton momenta $x>0.1$ is reduced, with the 3D dijet data providing tighter constraints at higher values of *x* compared to the 2D data. The strong coupling constant at the Z boson mass is determined simultaneously with the PDFs, yielding consistent results between the 2D and 3D dijet measurements, with the former resulting in the slightly more precise value of $\alpha _\textrm{S} (m_{{\text {Z}}}) = 0.1179 \pm 0.0019$ at NNLO.

The impact of subleading-color contributions to the leading-color NNLO calculation used here is not yet known [41]. Apart from being useful as inputs to PDF fits or studies of jet size dependence, the present 2D and 3D measurements for two jet size parameters, $R=0.4$ and 0.8, and for the two dijet observables $m_{1,2}$ and $\langle p_{\textrm{T}} \rangle _{1,2}$, provide an ideal testing ground for further investigations.

## Figures and Tables

**Fig. 13:**
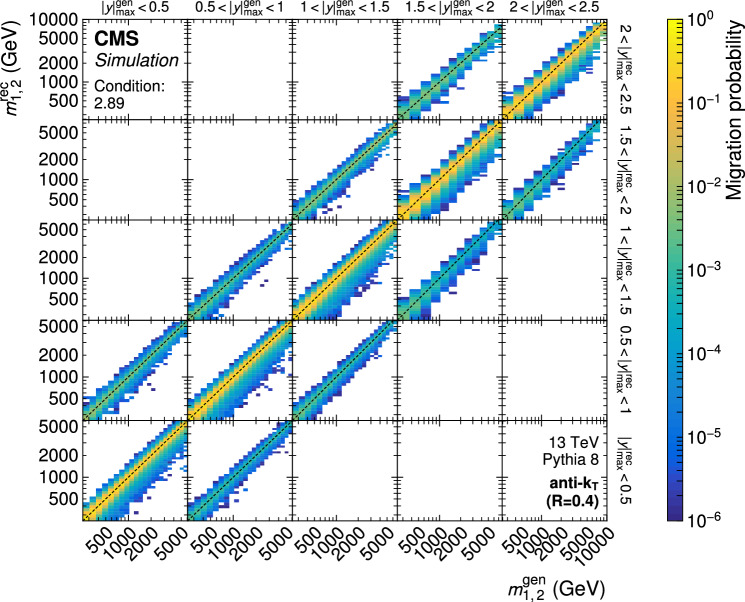
Response matrices for the 2D measurements as a function of $$|y |_{\text {max}} $$ and $$m_{1,2} $$ for jets with $$R = 0.4$$. The details correspond to those of Fig. 2

**Fig. 14:**
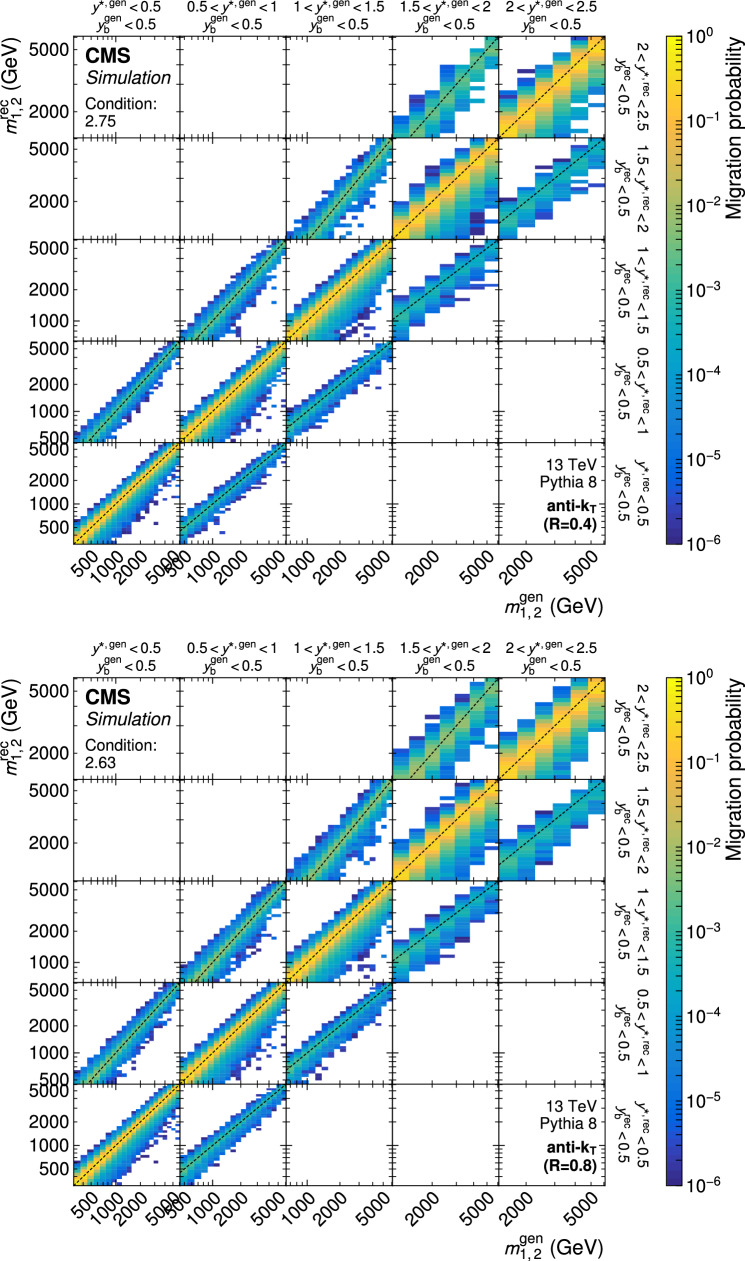
Partial response matrices for the 3D measurements as a function of $$m_{1,2} $$ using jets with $$R = 0.4$$ (upper) and 0.8 (lower), shown here for the five rapidity regions with $$y_{\text {b}} < 0.5$$. The details correspond to those of Fig. 2

**Fig. 15:**
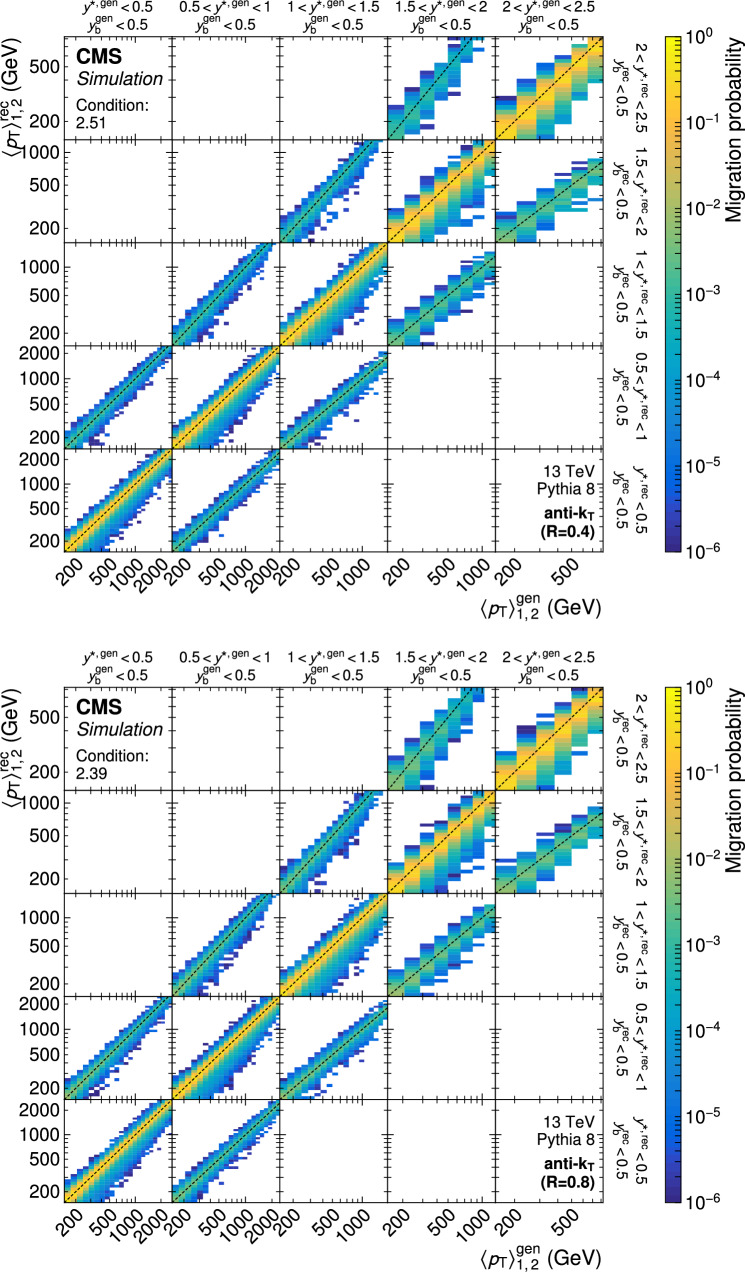
Partial response matrices for the 3D measurements as a function of $\langle p_{\textrm{T}} \rangle _{1,2}$ using jets with $R = 0.4$ (upper) and 0.8 (lower), shown here for the five rapidity regions with $y_{\text {b}} < 0.5$. The details correspond to those of Fig. 2

**Fig. 16:**
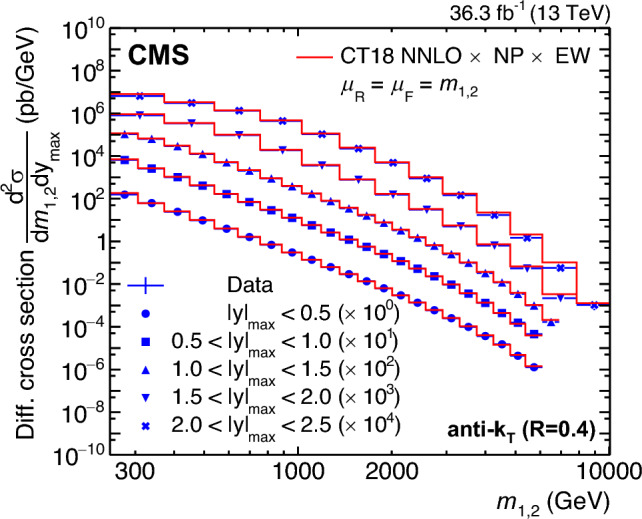
Overview of the 2D dijet cross section as a function of $$m_{1,2} $$ in all 5 $$|y |_{\text {max}} $$ regions, using jets with $$R = 0.4$$. The details correspond to those of Fig. 8

**Fig. 17:**
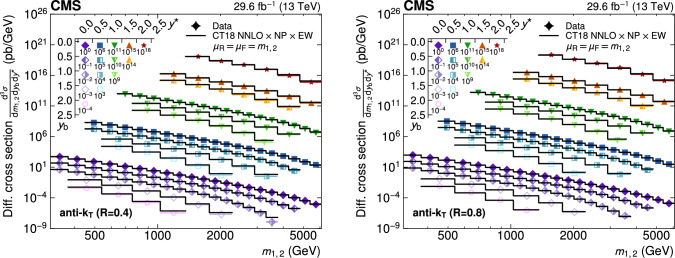
Overview of the 3D dijet cross section as a function of $$m_{1,2} $$ in all 15 $${(y^{*} \!,~y_{\text {b}})}$$ regions, using jets with $$R = 0.4$$ (left) and 0.8 (right). The details correspond to those of Fig. 8

**Fig. 18:**
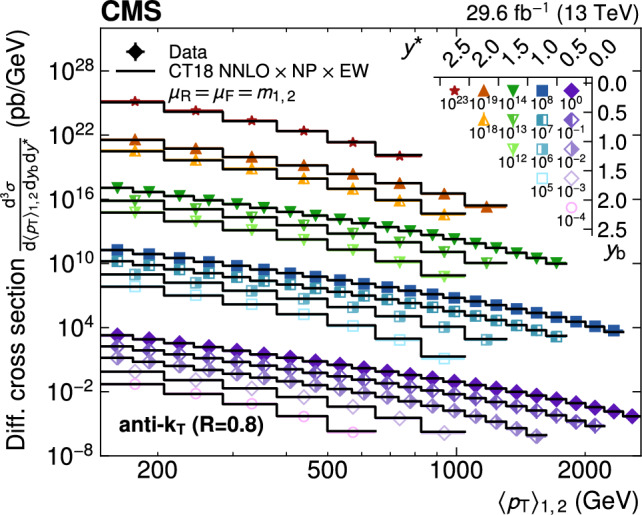
Overview of the 3D dijet cross section as a function of $\langle p_{\textrm{T}} \rangle _{1,2}$ in all 15 ${(y^{*} \!,~y_{\text {b}})}$ regions, using jets with $R = 0.8$. The details correspond to those of Fig. 8

**Fig. 19:**
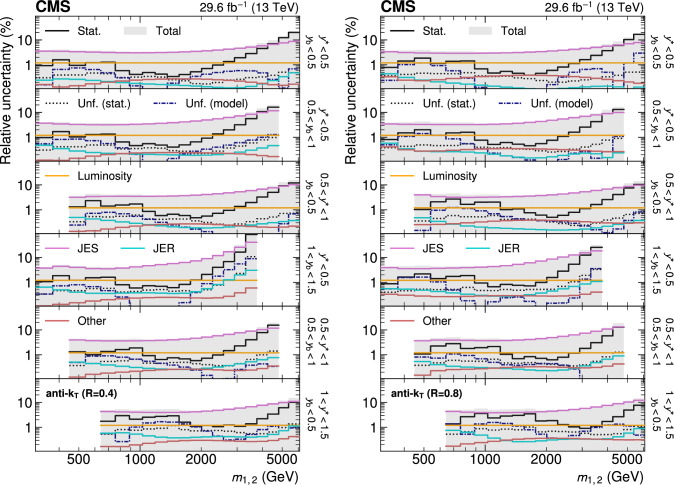
Breakdown of the experimental uncertainty for the 3D measurements as a function of $m_{1,2}$ using jets with $R = 0.4$ (left) and 0.8 (right), in six out of 15 ${(y^{*} \!,~y_{\text {b}})}$ bins. The details correspond to those of Fig. 4

**Fig. 20:**
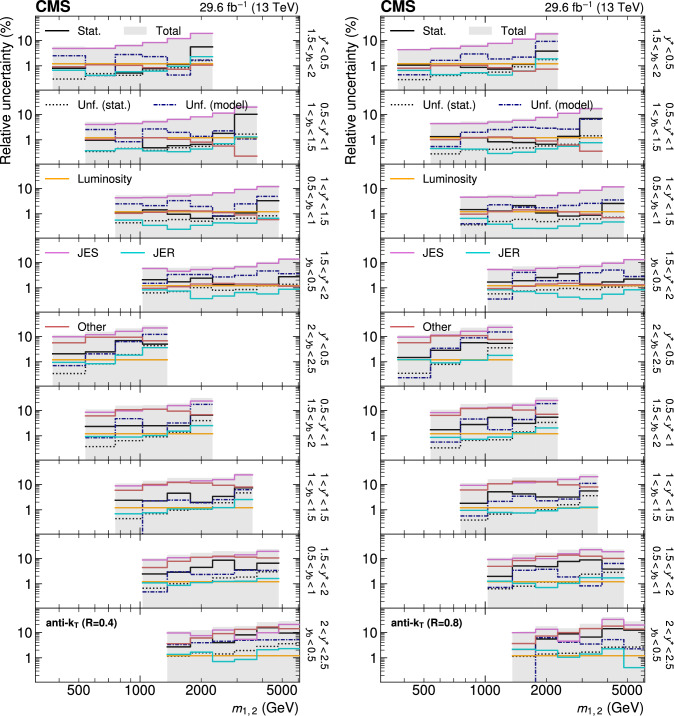
(*continuation of Fig.* 19) Breakdown of the experimental uncertainty for the 3D measurements as a function of $m_{1,2}$ using jets with $R = 0.4$ (left) and 0.8 (right), in the remaining nine out of 15 ${(y^{*} \!,~y_{\text {b}})}$ bins. The details correspond to those of Fig. 4

**Fig. 21:**
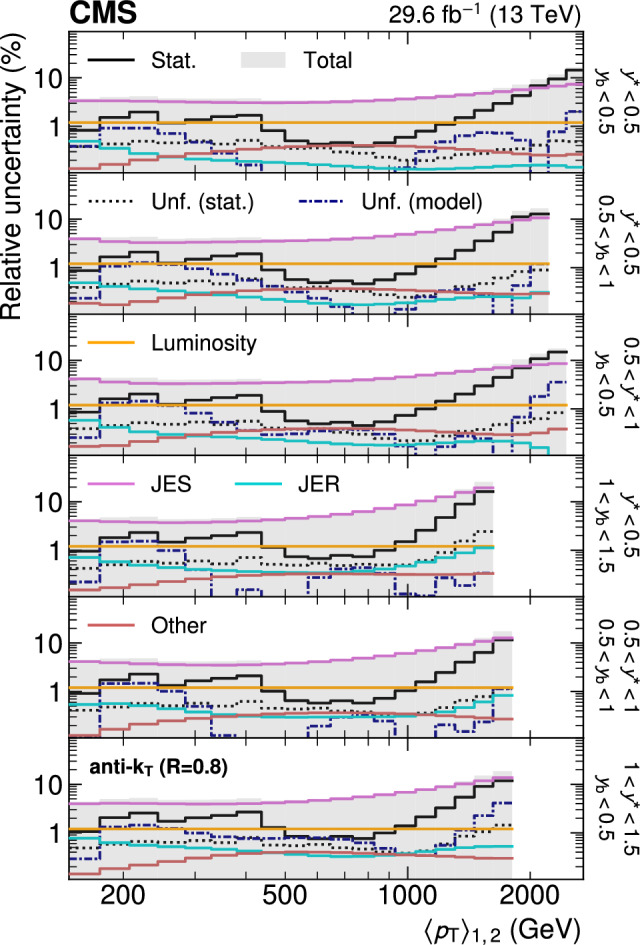
Breakdown of the experimental uncertainty for the 3D measurements as a function of $\langle p_{\textrm{T}} \rangle _{1,2}$ using jets with $R = 0.8$, in six out of 15 ${(y^{*} \!,~y_{\text {b}})}$ bins. The details correspond to those of Fig. 4

**Fig. 22:**
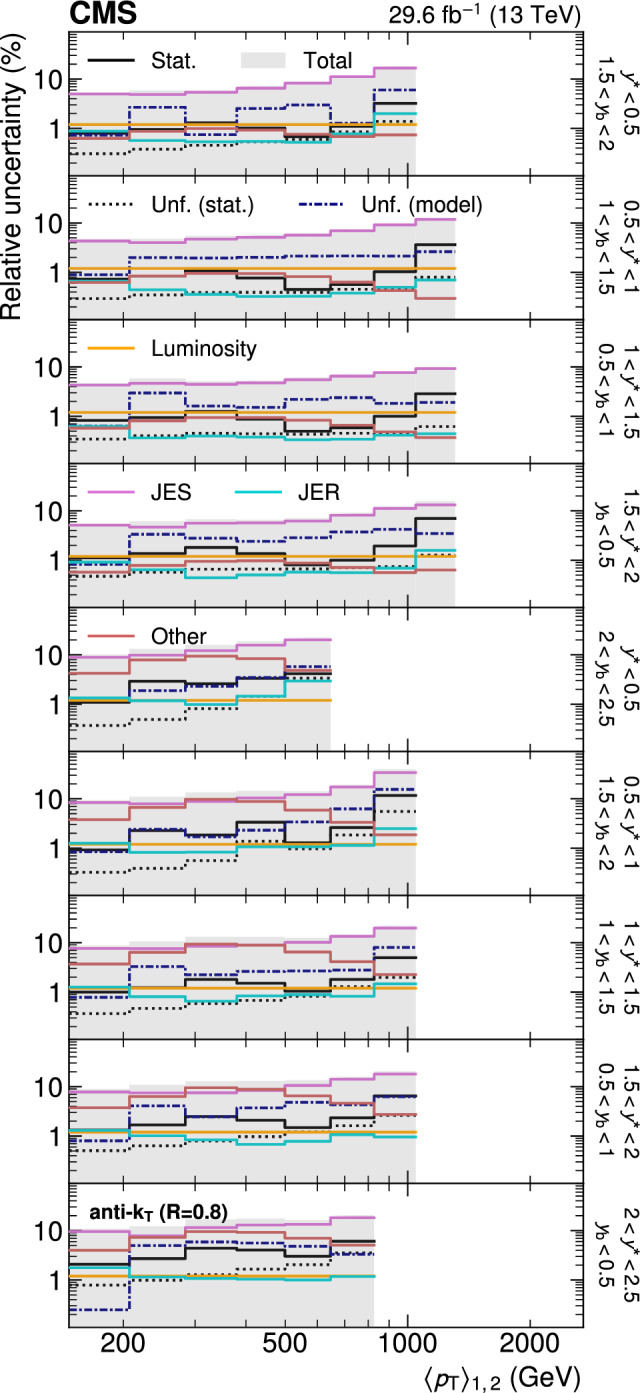
(*continuation of Fig.* 21) Breakdown of the experimental uncertainty for the 3D measurements as a function of $\langle p_{\textrm{T}} \rangle _{1,2}$ using jets with $R = 0.8$, in the remaining nine out of 15 ${(y^{*} \!,~y_{\text {b}})}$ bins. The details correspond to those of Fig. 4

**Fig. 23:**
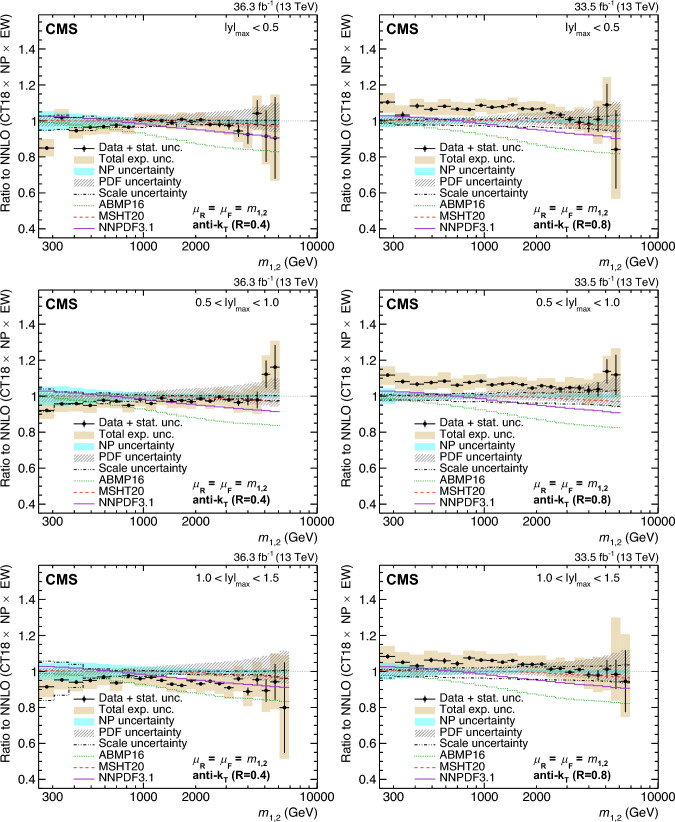
Comparison of the 2D dijet cross section for jets with $$R = 0.4$$ (left) and 0.8 (right) as a function of $$m_{1,2} $$ to fixed-order theoretical calculations at NNLO, shown here for three inner $$|y |_{\text {max}} $$ regions. The details correspond to those of Fig. 9

**Fig. 24:**
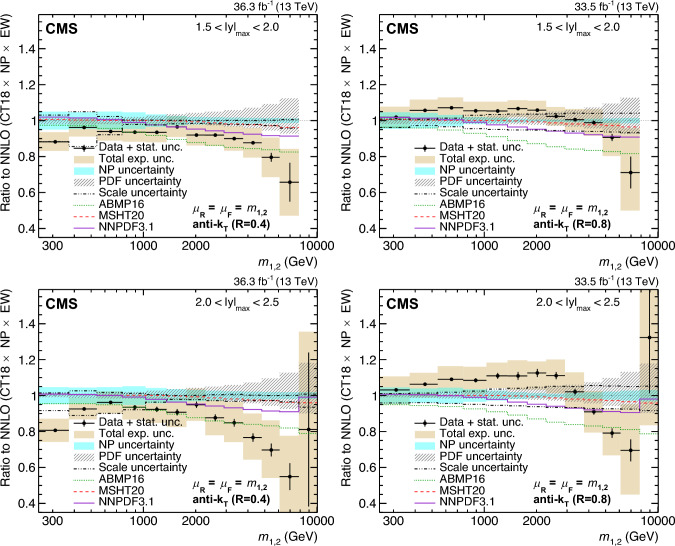
(*continuation of Fig.* 23) Comparison of the 2D dijet cross section for jets with $$R = 0.4$$ (left) and 0.8 (right) as a function of $$m_{1,2} $$ to fixed-order theoretical calculations at NNLO, shown here for two outermost $$|y |_{\text {max}} $$ regions. The details correspond to those of Fig. 9

**Fig. 25:**
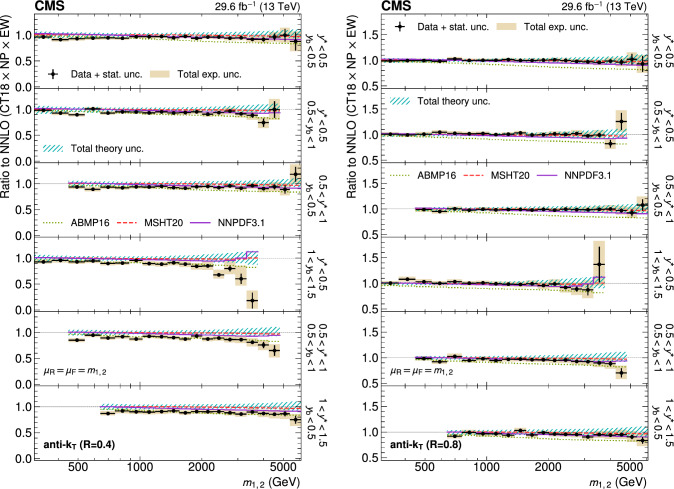
Comparison of the 3D dijet cross section as a function of $$m_{1,2} $$ to fixed-order theoretical calculations at NNLO, using jets with $$R = 0.4$$ (left) and 0.8 (right), in six out of the total 15 $${(y^{*} \!,~y_{\text {b}})}$$ bins. The details correspond to those of Fig. 10

**Fig. 26:**
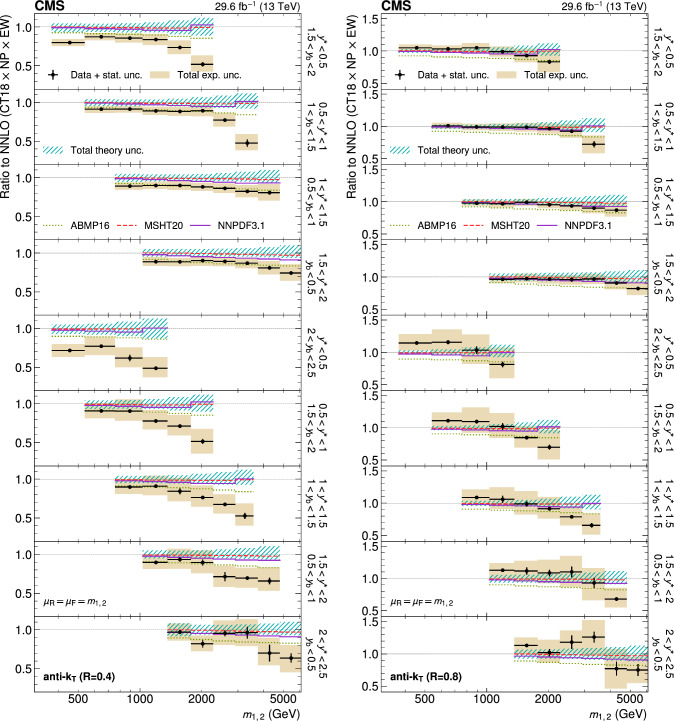
(*continuation of Fig.* 25) Comparison of the 3D dijet cross section as a function of $$m_{1,2} $$ to fixed-order theoretical calculations at NNLO, using jets with $$R = 0.4$$ (left) and 0.8 (right), in the remaining nine out of 15 $${(y^{*} \!,~y_{\text {b}})}$$ bins. The details correspond to those of Fig. 10

**Fig. 27:**
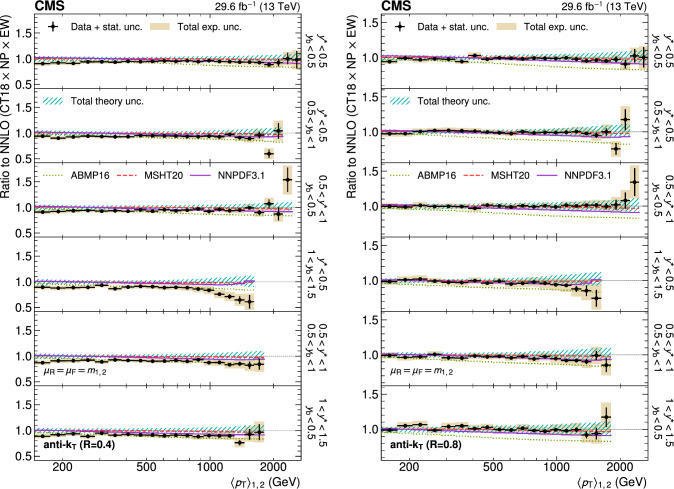
Comparison of the 3D dijet cross section as a function of $\langle p_{\textrm{T}} \rangle _{1,2}$ to fixed-order theoretical calculations at NNLO, using jets with $R = 0.4$ (left) and 0.8 (right), in six out of the total 15 ${(y^{*} \!,~y_{\text {b}})}$ bins. The details correspond to those of Fig. 10

**Fig. 28:**
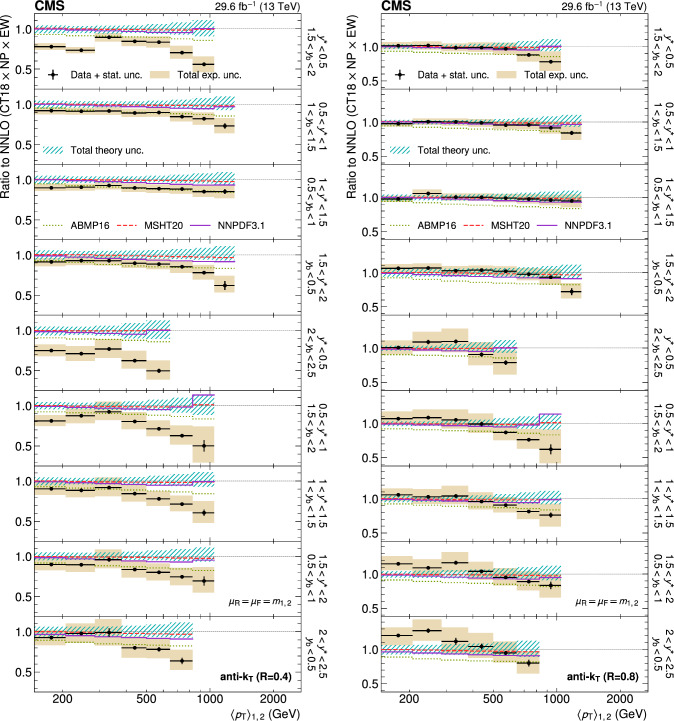
(*continuation of Fig.* 27) Comparison of the 3D dijet cross section as a function of $\langle p_{\textrm{T}} \rangle _{1,2}$ to fixed-order theoretical calculations at NNLO, using jets with $R = 0.4$ (left) and 0.8 (right), in the remaining nine out of 15 ${(y^{*} \!,~y_{\text {b}})}$ bins. The details correspond to those of Fig. 10

## Data Availability

Release and preservation of data used by the CMS Collaboration as the basis for publications is guided by the CMS data preservation, re-use and open access policy.

## Appendix A: Full-rapidity fit results

This section documents the results obtained for the fits described in Sect. 10 when all dijet rapidity regions are included. The partial $\chi ^2$ values indicating the goodness-of-fit in each rapidity region are given in Table 4, and Eqs. (11) and (12) show the values of $\alpha _\textrm{S} (m_{{\text {Z}}})$ obtained in fits including the 2D and 3D dijet cross sections, respectively:

**Table 4:** Goodness-of-fit values for the fits to the HERA DIS data alone, and together with the CMS dijet measurements, including all rapidity regions. The table shows the partial $\chi ^2$ values divided by the number of data points for the HERA DIS datasets and each of the dijet rapidity regions. The total $\chi ^2$ value, divided by the number of degrees of freedom, is given at the bottom of the table

		Partial $\chi ^2$ / $n_\text {data}$	
		HERA DIS + CMS 13$\,\text {Te}\hspace{-.08em}\text {V}$ dijets	
Data set		2D	3D
CMS dijet 2D			
$|y |_{\text {max}} < 0.5$		24 / 22	
$0.5< |y |_{\text {max}} < 1$		14 / 22	
$1< |y |_{\text {max}} < 1.5$		22 / 23	
$1.5< |y |_{\text {max}} < 2$		15 / 12	
$2< |y |_{\text {max}} < 2.5$		30 / 12	
CMS dijet 3D			
$y_{\text {b}} < 0.5$,	$y^{*} < 0.5$		32 / 21
$y_{\text {b}} < 0.5$,	$0.5< y^{*} < 1$		23 / 19
$0.5< y_{\text {b}} < 1$,	$y^{*} < 0.5$		40 / 19
$y_{\text {b}} < 0.5$,	$1< y^{*} < 1.5$		45 / 17
$0.5< y_{\text {b}} < 1$,	$0.5< y^{*} < 1$		18 / 17
$1< y_{\text {b}} < 1.5$,	$y^{*} < 0.5$		44 / 17
$y_{\text {b}} < 0.5$,	$1.5< y^{*} < 2$		15 / 7
$0.5< y_{\text {b}} < 1$,	$1< y^{*} < 1.5$		7 / 7
$1< y_{\text {b}} < 1.5$,	$0.5< y^{*} < 1$		9 / 7
$1.5< y_{\text {b}} < 2$,	$y^{*} < 0.5$		20 / 6
$y_{\text {b}} < 0.5$,	$2< y^{*} < 2.5$		19 / 6
$0.5< y_{\text {b}} < 1$,	$1.5< y^{*} < 2$		16 / 6
$1< y_{\text {b}} < 1.5$,	$1< y^{*} < 1.5$		6 / 6
$1.5< y_{\text {b}} < 2$,	$0.5< y^{*} < 1$		1 / 5
$2< y_{\text {b}} < 2.5$,	$y^{*} < 0.5$		15 / 4
HERA1+2			
CC ${\text {e}} ^{-} {\text {p}}$, $E_\text {p} = 920\,\text {Ge}\hspace{-.08em}\text {V}$		50 / 42	48 / 42
CC ${\text {e}} ^{+} {\text {p}}$, $E_\text {p} = 920\,\text {Ge}\hspace{-.08em}\text {V}$		37 / 39	41 / 39
NC ${\text {e}} ^{-} {\text {p}}$, $E_\text {p} = 920\,\text {Ge}\hspace{-.08em}\text {V}$		222 / 159	227 / 159
NC ${\text {e}} ^{+} {\text {p}}$, $E_\text {p} = 460\,\text {Ge}\hspace{-.08em}\text {V}$		197 / 177	201 / 177
NC ${\text {e}} ^{+} {\text {p}}$, $E_\text {p} = 575\,\text {Ge}\hspace{-.08em}\text {V}$		186 / 221	187 / 221
NC ${\text {e}} ^{+} {\text {p}}$, $E_\text {p} = 820\,\text {Ge}\hspace{-.08em}\text {V}$		55 / 61	55 / 61
NC ${\text {e}} ^{+} {\text {p}}$, $E_\text {p} = 920\,\text {Ge}\hspace{-.08em}\text {V}$		368 / 317	365 / 317
Total $\chi ^2/n_\text {dof}$		1283 / 1094	1557 / 1167

## Appendix B: Additional figures

See Figs. 13, 14, 15, 16, 17, 18, 19, 20, 21, 22, 23, 24, 25, 26, 27 and 28.

## Code Availability

The CMS core software is publicly available on GitHub (https://github.com/cms-sw/cmssw).
